# Oversight asymmetry in AI-assisted clinical documentation: a comparative-abductive study using neonatal discharge as a test context

**DOI:** 10.3389/fdgth.2026.1986499

**Published:** 2026-10-28

**Authors:** Samuel Oluwatobi Atiku, Olufisayo Olakotan, Kehinde Agbaje, Kehinde Owolanke, Thillagavathie Pillay

**Affiliations:** 1 Digital Technology and Innovation, University of Staffordshire, United Kingdom; 2 Research Services, Aston University Birmingham, United Kingdom; 3 Department of Neonatology, Women and Children’s Directorate, University Hospitals of Leicester NHS Trust, Leicester, United Kingdom; 4 Faculty of Science and Engineering, University of Wolverhampton, Wolverhampton, United Kingdom; 5 University of Suffolk, United Kingdom; 6 Department of Obstetrics and Gynaecology, Mersey and West Lancashire Teaching Hospitals NHS Trust, United Kingdom; 7 College of Health Sciences, University of Leicester, Leicester, United Kingdom

**Keywords:** artificial intelligence, clinical documentation, digital paediatrics, healthcare governance, human oversight, neonatal discharge, patient safety, qualitative research

## Abstract

**Background:**

Human oversight is widely treated as a safeguard for clinical artificial intelligence (AI), yet a reviewer may remain formally responsible without having the knowledge, time, or authority required to verify generated documentation. We develop “oversight asymmetry” as a prospective governance diagnosis and use neonatal discharge as a theoretical test context for responsible digital paediatrics, not as an observed setting of AI use.

**Methods:**

We conducted a comparative-abductive, netnographically informed qualitative study of 64 public Reddit threads posted between 2021 and April 2026, with comment trees retrieved under pre-specified extraction parameters. The threads contained 2,848 comments and replies from 1,723 pseudonymised accounts in nursing, neonatal, paediatric, medicine, and health informatics communities, and 49 threads contributed to the final themes. A discharge and transition-home stream, drawn mainly from general nursing communities, and a smaller AI documentation and communication stream were analysed separately using reflexive thematic analysis before abductive comparison. Professional role was attributed only where contributors explicitly self-positioned.

**Results:**

Discharge readiness was described as depending on knowledge distributed across records, professionals, bedside observation, and families, while responsibility for release could become concentrated. Frontline responsibility was separable from institutional authority, although several accounts described distributed oversight that worked because escalation was available. In four anchor threads from two communities, AI documentation discussions described faster drafting alongside residual source checking and correction, and a narrower set of accounts described reconstruction of missing clinical rationale. Workload accounts, together with AI-linked predictions in two threads, suggest that anticipated efficiency may be converted into higher workload before residual verification work is removed. We propose that oversight becomes vulnerable when verification responsibility and demand exceed epistemic access, protected time or consequential authority.

**Conclusion:**

Oversight asymmetry identifies a prospective mechanism through which nominal human review may fail to constitute meaningful oversight. Responsible paediatric artificial intelligence governance should measure residual verification work, preserve provenance and access to family and bedside context, protect review time, and provide effective output-level and system-level escalation authority before automation is treated as additional capacity. The study generates propositions for prospective evaluation and does not claim that artificial intelligence-assisted neonatal discharge was directly observed.

## Introduction

Human oversight has emerged as a prominent governance response to risks associated with healthcare artificial intelligence (AI) (1–4). The World Health Organization states that humans should remain in control of healthcare systems and medical decisions (4). The European Union Artificial Intelligence Act establishes binding human oversight requirements for high-risk AI systems. Generative clinical documentation systems are not classified as high risk merely because they generate clinical text, although a documentation tool that qualifies as a medical device requiring notified body conformity assessment under the Medical Device Regulation falls within the high-risk category through Article 6(1) (1). Article 14 requires persons assigned to oversight of high-risk systems to understand relevant capabilities and limitations, remain alert to automation bias, interpret outputs, and be able to disregard, override, or interrupt the system where appropriate (1). Meaningful oversight is therefore concerned with what a reviewer is practically equipped to know and do, rather than the mere formal retention of a human decision point (2, 3).

This distinction is consequential in generative clinical documentation. AI-generated discharge documents and note summaries can be readable, concise, and coherent while still containing omissions, hallucinations, or clinically relevant inaccuracies (5–8). Studies on ambient AI scribes report reductions in documentation time or cognitive burden, but clinicians continue to describe editing requirements and concerns about accuracy, completeness and note construction (9–11). Rewriting clinical notes with generative AI can likewise improve linguistic presentation while losing medically relevant detail (12). Consequently, documentation assistance may reduce some production work without removing the substantive work required to verify whether generated text is clinically faithful (13–15).

The governance significance of this residual work is tied to verification demand. Verification complexity concerns the cognitive effort required to determine whether automated assistance is functioning correctly, and higher complexity can impair detection of automation failures (16). Automation bias research and experimental studies also show that incorrect automated advice can influence professional judgement, while explanatory features do not consistently produce appropriately calibrated trust (17–19).

Reviews of healthcare AI use identify time, competing priorities, data access, understanding, professional autonomy, and organisational readiness as conditions shaping whether clinicians can use AI safely and effectively (20–22). Recent healthcare-specific oversight analyses similarly question clinician-in-the-loop arrangements where responsibility is retained by clinicians without corresponding epistemic, organisational, or decisional capacity (3, 23, 24). Human oversight in AI-assisted documentation may therefore become an allocation problem where responsibility for verification exceeds the information, time, or authority practically available to the reviewer.

Current scholarship identifies these operational constraints, but prospective tools for diagnosing the mismatch between assigned verification duties and practical clinical capacities remain limited. This study addresses that gap by defining the imbalance between verification demand and a clinician's practical ability to verify artificial intelligence-generated records safely.

To diagnose this organisational mismatch, this paper defines “oversight asymmetry” as a condition where a specific practitioner is expected to verify and correct an AI output, but lacks the time, knowledge, or institutional authority to do so effectively. The concept therefore focuses on whether the assigned responsibility matches the reviewer's practical capacity, rather than merely confirming that a human is formally present in the workflow.

Compared with Meaningful Human Control and responsibility gap frameworks, oversight asymmetry has a narrower, prospective focus. Existing work examines accountability across human–machine systems and the concentration of liability around human operators after failures (2, 25–28). Oversight asymmetry instead diagnoses whether an identified reviewer has the practical capacity to perform required checking before an error occurs. This is consistent with healthcare artificial intelligence research showing that clinician presence does not constitute genuine oversight when cognitive space, knowledge, or institutional support is inadequate (3, 23, 24). The closest existing account is the meaningful-oversight model of van de Sande and colleagues, which treats epistemic capacity, cognitive space, decisional authority, and intervention effectiveness as interlocking conditions of oversight (3). Oversight asymmetry draws on those conditions but differs in its unit of analysis. It compares one assigned verification obligation, and the demand that obligation creates, with the capacities of the particular reviewer who holds it, so the same capacities may be adequate for one task and insufficient for another. Table 1 presents how the concept differs from these neighbouring accounts.

**Table 1:** Oversight asymmetry compared with neighbouring accounts of human control and responsibility.

Account	Unit of analysis	Timing	Central question	Diagnostic condition	Governance response
Meaningful human control (2, 25)	Human–machine system	Design and use, largely normative	Do humans retain control commensurate with their responsibility?	Tracking or tracing conditions are unmet	Design systems so outcomes remain traceable to human reasons
Meaningful oversight beyond the human in the loop (3)	Oversight arrangement for a medical AI system	Deployment and use	Are epistemic capacity, cognitive space, decisional authority, and intervention effectiveness present?	One or more conditions is absent	Build the four conditions into deployment
Responsibility gaps (28)	Distributed human–machine agency	Mostly retrospective	Can culpability, accountability, or active responsibility be attributed?	Responsibility is missing or indeterminate	Redesign roles and institutions to close the gap
Moral crumple zones and liability sinks (26, 27)	Human operator within a sociotechnical system	Retrospective, after failure	Where do blame and liability settle?	Blame settles on an operator with limited control	Redistribute liability and recognise system contribution
Oversight asymmetry (this study)	A named reviewer and one specific verification obligation	Prospective, before and during deployment	Can this reviewer perform this checking with the information, time, and authority actually available?	A consequential capacity deficit relative to the assigned obligation and its demand	Measure residual verification, protect review time, and provide output-level and system-level authority before counting savings

Diagnosis rests on the relationship among five elements rather than on any one of them. These are the responsibility assigned to the reviewer, the cognitive and practical burden of verification, access to the records and contextual knowledge needed to verify the output, the practical time available, and the authority to make a detected problem consequential at output or system level. The relationship is directional but not quantitative, and the elements are not assumed to be commensurable or additive. Oversight asymmetry is supported where a substantive verification obligation is assigned, at least one relevant capacity is materially insufficient relative to that obligation, and the deficit constrains detection, correction, escalation, or justified finalisation. A single capacity deficit may therefore be sufficient when it is consequential, whereas several deficits may coexist. Conversely, an AI error, workload complaint, professional disagreement, or requirement to check is not evidence of asymmetry on its own, and high responsibility is not asymmetric when verification demand is manageable and adequate information, time, and authority are available. The same task may therefore be proportionate in one setting and asymmetric in another.

One implication is contingent transparency. Provenance, source links, and explanations may improve oversight when they reduce verification demand for a reviewer who has the expertise, time, and authority to act, but transparency does not compensate for missing capacity elsewhere (19, 21). Recent healthcare oversight work likewise treats information access, decisional authority, and workable intervention routes as jointly relevant to meaningful oversight (3). Transparency is therefore treated as a condition affecting the responsibility–capacity relationship rather than an independent guarantee of safe human control.

Responsible digital paediatrics must account for settings in which clinically relevant knowledge is distributed across time, professionals, and families rather than contained in a single record. To examine how this matters for human oversight, this study analyses neonatal discharge as a comparative test case rather than claiming to observe a deployed artificial intelligence system. Readiness for home transition develops through continuing medical stabilisation, caregiver education, and team coordination, which makes neonatal discharge a demanding context in which to test what substantive verification of an automated clinical representation would require (29–32).

Discharge preparation in the neonatal intensive care unit (NICU) involves medical history, treatment planning, caregiver competence, continuing education, and coordination across professional and family actors (30, 32, 33). Nursing work is central to safe paediatric discharge through education, communication, coordination, and assessment of family capability (31, 34). The knowledge relevant to transition readiness is therefore distributed across formal records, multidisciplinary judgements, repeated bedside observations, and caregiver learning rather than generated at one documentary moment. These features connect neonatal discharge to person-centred fundamental care, which integrates physical, psychosocial, and relational needs within the context in which care is delivered (35, 36). Safe transition depends not simply upon documentary completeness, but upon communication, caregiver education, relational knowledge, coordination, and responsiveness to individual and family capability. In technology-supported workflows, the relevant question is whether artificial intelligence assists these practices without obscuring or displacing the contextual knowledge through which nurses, other professionals, and families establish readiness for discharge.

If generative artificial intelligence is used to summarise or draft neonatal discharge documentation, substantive verification would require access to this distributed and relational history. The neonatal stream therefore supplies evidence about the organisation of knowledge and responsibility in a paediatric transition, whereas direct artificial intelligence documentation discussions supply evidence about verification work. Their comparison is intended to inform governance for responsible digital paediatrics, particularly where responsibility for finalising an automated representation is concentrated on one actor while the knowledge, time, or authority needed to verify it remains dispersed.

The research question was as follows: How do online nursing and related professional communities construct responsibility, verification capacity, and authority across neonatal discharge and artificial intelligence-assisted clinical documentation or communication, and what do these accounts suggest about governance conditions for responsible digital paediatrics when oversight asymmetry may arise?

## Materials and methods

### Research design and analytical orientation

We used a comparative-abductive, netnographically informed qualitative design combining computational retrieval of public Reddit discussions with thread-level reflexive thematic analysis. Netnography preserved interactional context, reflexive thematic analysis supported patterned interpretation, and abductive comparison related data-near patterns to theory-informed explanation (37–39). The study developed theory rather than evaluating a deployed neonatal AI system. Three empirically distinct streams were retained: a discharge and transition-home stream concerning readiness knowledge, workload, responsibility, and authority in neonatal, maternal–newborn, and some general adult settings; AI-specific material concerning generated documentation, verification, and communication; and one healthcare AI governance thread providing contextual material. Cross-stream comparison followed within-stream interpretation, so the resulting claims are abductive propositions rather than observations of AI-assisted neonatal discharge.

The retrieved Reddit thread was the primary analytic unit. Initiating posts and nested replies were retained together so that agreement, challenge, and qualification remained interpretable within their conversational sequence (38, 40–42). Computational procedures supported retrieval, duplicate control, hierarchy reconstruction, and auditability, while coding and interpretation remained qualitative.

Analysis used a contextualist and interpretivist orientation. Researcher interpretation was treated as part of reflexive thematic analysis, so intercoder reliability was not calculated (37, 43). Reddit contributions were interpreted as situated accounts rather than verified descriptions of staffing, institutional policy, or clinical practice, and professional role or jurisdiction was used only where contributors explicitly self-positioned. Analytic memos distinguished direct empirical findings within an evidence stream, cross-stream comparisons, and abductive propositions about conditions under which oversight asymmetry may arise. The final level is theory development rather than evidence that compared conditions that co-occurred in the same workflow (39, 44).

### Role of oversight asymmetry in the analysis

We did not use oversight asymmetry as an eligibility criterion, search term, or predetermined coding template. Requiring terms such as “oversight,” “accountability,” “verification,” or “authority” at retrieval would have favoured discussions already framed in language compatible with the proposed concept. We therefore began with predominantly inductive familiarisation and first-cycle coding.

Only after provisional patterns and candidate themes had been developed did we interrogate them abductively through five sensitising dimensions, assigned responsibility, verification demand, epistemic capacity, temporal capacity, and institutional authority. Sensitising concepts can guide attention without functioning as fixed categories that predetermine what must be found (45). The dimensions were therefore interpretive prompts rather than measured variables or compulsory codes. The analysis moved from data-near descriptions towards theory-informed explanation through iterative comparison rather than from a fixed framework towards confirming examples (39, 44). This sequencing was also consistent with related sociotechnical netnography in which open coding, comparison, and analytic memoing preceded higher-order theoretical organisation (40). The five sensitising dimensions and their analytical questions are summarised in Table 2.

**Table 2:** Sensitising dimensions used during abductive analysis.

Sensitising dimension	Analytical question
Assigned responsibility	Who was expected to detect, check, correct, explain, sign, escalate, or answer for the information or outcome?
Verification demand	What work was required to establish that an output, document, or discharge representation was adequate?
Epistemic capacity	Could the responsible actor access the records, observations, contextual knowledge, or reasoning required for that judgement?
Temporal capacity	Was sufficient practical opportunity available to undertake the required checking or reconciliation?
Institutional authority	Could the actor amend, reject, delay, escalate, or otherwise make an identified problem consequential?

We also applied an explicit negative-case rule. An AI error, workload complaint, difficult discharge, or requirement for checking was insufficient on its own to constitute oversight asymmetry. We examined whether an identifiable responsibility was constructed as disproportionate to the information, time, or authority available to discharge it. Accounts describing adequate source access, manageable verification, sufficient time or effective authority were retained as counterexamples and used to refine the boundaries of the interpretation. The rule was also applied to authority. Where an actor lacked final decision rights but described an effective route for escalation, the arrangement was read as distributed oversight rather than asymmetry, and it became asymmetric only where a capacity deficit prevented a recognised problem from being detected, corrected, or escalated.

### Data source, search strategy, and computational retrieval

We searched 16 predefined Reddit communities spanning nursing, neonatal, paediatric, medicine, health informatics, and wider health-related discussion (Supplementary Table S1). Eight communities returned candidate material, and five contributed retained threads (r/nursing, r/medicine, r/NICU, r/pediatrics, and r/healthIT). Community selection was purposive and intended to provide analytic range rather than statistical representativeness. Reddit was selected because threaded online discussion preserves asynchronous interaction in which contributors can elaborate upon, contest, and qualify one another's accounts, while methodological guidance cautions that such material must be interpreted within its platform and conversational context (42).

Data were retrieved through Reddit's Data API using the Python Reddit API Wrapper, PRAW, through a script application registered in October 2025. When Reddit closed self-service API access in November 2025, it stated that existing access would not be affected, and the application continued to operate under Reddit's non-commercial Data API terms throughout data collection. Data were collected in stages between 3 March 2026 and 16 May 2026, and coding followed data collection. Each query was run within each target community with relevance sorting and no time filter. Candidate submissions returned by more than one query or community were deduplicated using the Reddit submission identifier as the thread key, so a discussion could enter the candidate set only once. The latest retained thread was posted on 25 April 2026. In June 2026, after data collection had ended, Reddit updated its Responsible Builder Policy to direct academic research through its Reddit for Researchers programme (46), so a replication of this design would now require access through that programme.

The pipeline imposed a maximum of 250 returned submissions for each query, a ceiling of 900 comments per thread and a replace_more() limit of 3. Because replace_more() could leave deeper branches unexpanded, the completeness of any thread cannot be established, and the material is described throughout as retrieved rather than complete. Comments whose author account had been deleted kept their text but carried no account identifier. They were analysed as text, pooled under a single placeholder, and excluded from counts of distinct accounts. The community frame, extraction limits, full vocabulary, and the exact 90 query strings are reported in Supplementary Tables S1, S2. Reddit score, search position, and voting metrics were not used as screening criteria or as evidential weight.

The search strategy combined four concept families. Generic AI expressions were linked with documentation terminology to reduce retrieval of unrelated consumer–AI discussions. Supplementary Table S2 reproduces the vocabulary for each concept family.

Ninety query strings were applied in each of the 16 target communities. The set was generated by quota rather than truncated from a longer list, and it comprised 48 combinations of AI, neonatal, and discharge terms, 18 direct AI documentation phrases, 12 vendor and product names, six generic AI documentation phrases, and six short neonatal AI phrases. The search vocabulary and extraction parameters were documented before substantive thematic interpretation, and no later query was added because it appeared likely to support oversight asymmetry. Reddit search appears to match terms loosely rather than requiring every term to be present. Many candidates for that reason carried neonatal or discharge vocabulary without any AI vocabulary, and the term premature retrieved discussion of early adult discharge as well as preterm infants. These effects were handled at screening and are reported as a limitation.

### Thread-level screening and corpus construction

We screened candidate material at the level of the retrieved thread rather than the title or initiating post alone. Eligibility decisions were made against the explicit inclusion and exclusion criteria reported in the Supplementary Material (Supplementary Table S3), while the initiating submission, top-level comments, and nested replies were read together because substantive relevance could arise later in the comment tree. Whole-thread reading is consistent with approaches that treat online discussion as interactional material rather than a collection of independent posts (38, 40–42). Keyword presence therefore generated candidates but did not determine eligibility. Borderline cases were resolved through reflective reading of whether the conversation substantively addressed documentation, communication, discharge, responsibility, or governance, and uncertainty was retained in the audit trail rather than converted into eligibility through lexical matching.

After deduplication, the computational search returned 97 candidate threads containing 3,823 retrievable comments and replies from 2,280 pseudonymised commenting accounts. The median candidate thread contained 10 comments and replies, the largest contained 491, and the deepest observed reply chain extended to 15 levels. This supported preservation of conversational hierarchy rather than flattening comments.

During screening, we found that the empirical domains were usually discussed separately. The study had begun with a narrower question about AI-assisted neonatal discharge documentation and an initial rule that a retained thread should address AI documentation, neonatal care, and discharge together. Read at thread level, almost no candidate discussion met that rule substantively. Where AI and newborn care did co-occur, the AI was used by parents or was a prospective hope rather than a documentation tool in neonatal discharge. Retaining only such threads would have produced a very small corpus and excluded the material needed to examine the responsibility–capacity problem.

We therefore revised the research question and eligibility criteria after candidate retrieval and initial screening, before coding began. Under the expanded problem-space strategy, threads were eligible where the retrieved discussion substantively addressed at least one of six areas relevant to the revised comparative research question. These comprised AI-assisted clinical documentation, AI use in discharge or clinical communication, patient or family AI use affecting clinical communication, neonatal or postpartum discharge and transition-home work, professional responsibility or workload around documentation and discharge, and directly relevant healthcare AI governance. We excluded material where relevance was incidental, where “AI” referred to a non-artificial intelligence meaning, where discussion was predominantly career-related, where no substantive connection to documentation, communication, discharge, or responsibility was present, or where insufficient material was retrievable for interpretation.

Reflective screening retained 64 of the 97 candidate threads, containing 2,848 comments and replies from 1,723 pseudonymised accounts. A later audit of the retained set found that 15 of these threads made no contribution to the final themes. Eight were career-focused and seven concerned general occupational experience without a substantive link to discharge, documentation, or authority, so under the stated criteria they should have been excluded at screening. They supplied no extract and no thematic evidence, and they are reported rather than silently removed so that all counts remain traceable. The 49 contributory threads contained 2,473 comments and replies. Three contributory threads had no retrievable replies (T40, T58, and T60) and informed the analysis through their initiating posts alone. Each thread was allocated to one evidence stream according to its substantive subject, so that a discussion whose main subject was AI-mediated documentation or communication entered the AI stream even where newborn care was also discussed. The composition of the corpus is reported in Table 3, the study flow in Supplementary Figure S1 and a thread-level inventory in Supplementary Table S4.

**Table 3:** Retained corpus by evidence stream and contribution to final themes.

Evidence stream	Threads retained	Comments and replies	Contributory threads	Comments in contributory threads
Discharge and transition home (neonatal, maternal–newborn, paediatric and general discharge)	49	2,119	41	2,003
AI clinical documentation and communication	7	423	7	423
Healthcare AI governance	1	47	1	47
Retained at screening without stream allocation (non-contributory)	7	259	0	0
Total	64	2,848	49	2,473

The evidence streams remained distinguishable throughout analysis. Of the 49 discharge stream threads, 32 concerned NICU, newborn, postpartum, or labour and delivery settings, four concerned paediatric care, and 13 concerned adult or unspecified settings; the last group was read as a general discharge and workload comparator and is labelled as such in the Results. Discharge stream material was not treated as direct evidence that AI was affecting neonatal discharge, while AI discussions outside neonatal care were not treated as evidence of neonatal practice. The cross-stream evidential architecture and the status of the resulting abductive propositions are set out in Table 4.

**Table 4:** Cross-stream evidential architecture and abductive propositions.

Oversight dimension	Discharge and transition-home stream	AI documentation stream	Evidential status	Abductive proposition
Assigned responsibility	Readiness and discharge work are distributed, while sign-off, release, or escalation duties may become concentrated	Professional users describe responsibility for reading, correcting, and finalising generated material	Direct finding within each stream, comparison made across streams	Responsibility can be identifiable even when upstream work and knowledge remain distributed
Verification demand	Readiness requires longitudinal synthesis of observation, caregiver capability, coordination, and formal records	Generated summaries may require source checking, rationale recovery, and correction of omissions or errors	Discharge stream supplies contextual evidence about what substantive verification would need to recover, AI stream directly evidences verification work	A concise output may conceal rather than remove the work required to establish adequacy
Epistemic capacity	Relevant knowledge is distributed across bedside observation, families, services, and records	Checking depends on expertise, source access, and the ability to reconstruct omitted context	Cross-stream comparison only, this does not show that the same deficit occurred in neonatal AI use	Access to the final artefact is insufficient where the knowledge needed to verify it remains dispersed
Temporal capacity	Education, coordination, and discharge work compete with staffing and workload demands	Time savings may be absorbed by review, reconciliation, or higher throughput expectations	Direct findings on time pressure within each stream, linked through abductive inference	Automation can reduce production time without creating practical time for substantive oversight
Institutional authority	Frontline concern, refusal, or escalation may remain separate from final release authority	Users may edit a specific output while lacking authority over recurrent system defects, staffing assumptions, or deployment rules	Discharge stream authority pattern is direct, system-level AI proposition combines direct AI accounts with conceptual analysis	Output-level correction authority does not guarantee authority to remedy recurring conditions that generate verification burden

### Reflexive thematic analysis

We read each retained thread as a conversational case and prepared a structured analytic memo. Memos recorded the central issue being negotiated, actors to whom responsibility was assigned, forms of knowledge invoked, perceived constraints, authority claims, challenges, counterpositions, and preliminary interpretive questions. Individual comments functioned as embedded meaning units, but interpretation repeatedly returned to the surrounding thread, consistent with the study's netnographic and reflexive thematic orientation (38, 43).

First-cycle coding remained open and predominantly inductive. Working codes captured assigning, refusing, or sharing responsibility, unclear ownership, verification work, difficult-to-detect error, access to source information, information fragmentation, situated professional knowledge, caregiver knowledge, time pressure, verification as additional work, workarounds, amendment authority, restricted authority, escalation, professional scope, system-level control, and cases of adequate capacity. Codes were revised, combined, or discarded recursively and were not applied as a fixed reliability codebook, consistent with reflexive thematic analysis in which coding is an interpretive and evolving process (37, 43). Codes remained revisable throughout rather than being fixed at a single point. Boundary cases also constrained theme scope. A paediatric hospitalist discussion in which attending review was built into the staffing model (T40), a near-miss report handled through an investigation route (T46), and a thread in which a newborn was discharged while the mother remained an inpatient (T28) were retained as cases of workable distributed oversight, which narrowed Theme 2 from a general claim about hierarchy to a claim about consequential authority. The working codebook is provided in Supplementary Table S5.

Because the research question concerned how responsibility and authority were negotiated, we added an interactional coding layer. Replies were examined for agreement or reinforcement, challenge or rebuttal, qualification, responsibility redirection, knowledge challenge, authority claim, boundary defence, elaboration, workaround proposal, conversational settlement, continuing disagreement, and counterexample. This distinguished initiating claims from later replies that contested or qualified them and preserved the interactional context emphasised in online discussion research (38, 42).

Theme development proceeded recursively through coding, comparison, memoing, and return to whole threads, consistent with reflexive thematic analysis (37, 43). We compared patterned constructions within and across the evidence streams without collapsing their evidential differences. Candidate themes were reviewed against both coded extracts and whole threads, including contradictory cases. Only after provisional themes had stabilised were they examined through the five oversight-asymmetry dimensions. This abductive stage used theory to refine explanation without requiring each theme to correspond mechanically to one conceptual dimension.

### Reflexivity and analytic quality

Analytic quality was supported through structured memoing, repeated comparison of interpretations with whole threads, preservation of contradictory and negative cases, and an audit trail connecting retrieval, screening, coding, and theme development. The principal reflexive risk was circularity because oversight asymmetry was the sensitising concept being developed. To reduce that risk, it was excluded from retrieval and initial coding, first-cycle coding remained data-near, provisional themes were developed before abductive interpretation, and alternative explanations and adequate-capacity counter-cases were retained. Where researchers compared interpretations, disagreement prompted return to the relevant parent–child sequence and reconsideration of code or theme boundaries rather than calculation of intercoder reliability, consistent with reflexive thematic analysis (37, 43). Genuine ambiguity could remain as a qualification instead of being forced into consensus.

Reporting followed the Standards for Reporting Qualitative Research (47).

Researcher roles and reflexive governance: One researcher conducted the computational search and data retrieval. Two researchers jointly screened the candidate threads and coded the retained material, while two authors contributed to analysis and interpretation during theme refinement. Where interpretations differed, comparison was used to return to the relevant thread context, expose assumptions, and refine code or theme boundaries rather than to calculate intercoder reliability.

Researcher positionality: The team combined clinical, methodological, and informatics perspectives, and these shaped interpretation. Two authors work in neonatal services and a third in obstetrics and gynaecology, so the team brought practical familiarity with discharge readiness, parental teaching, and escalation within maternity and neonatal care. The lead author researches digital health and had previously published on AI scribes and AI-generated clinical notes (9, 40, 41). That prior work sensitised the team to verification burden, and it could have encouraged a reading of AI accounts as problematic by default. Two safeguards addressed the risk. Accounts of useful or proportionate AI assistance were coded as counter-cases rather than set aside, and interpretations of discharge material were checked with the authors working in neonatal and obstetric services so that assumptions carried over from the AI literature could be challenged against the thread itself.

We conducted a contributor concentration audit because Reddit participation was uneven. Leaving aside the placeholder that pooled comments from deleted accounts (209 comments across 31 threads, 7.3% of the total), the most prolific pseudonymised account contributed 2.6% of comments, the five largest contributors contributed 7.8%, and the 10 largest 10.2%. Candidate themes were checked for dispersion across supporting threads, contributors, evidence streams, and communities, with one-account-one-presence review where a pattern appeared to depend on repeated contributions. No theme depended on a single account. Across the anchor and supporting threads of each theme, the most prolific single account contributed between 2.8% and 9.4% of comments. The review did narrow Theme 3, because the claim about lost clinical rationale rested mainly on one initiating account and its replies. These quantities were diagnostic safeguards rather than prevalence measures, and a rich thread could remain analytically important without being written as a distributed pattern.

### Ethical handling of public online material

The study followed the University of Staffordshire Research Ethical Review Policy (amended December 2024). It qualified for the Level 1 Ethics Disclaimer route for research using information freely available in the public domain, and the disclaimer form (reference DEF-A041261N-001) was completed before data collection, so proportionate or full ethical review was not required. No contributor was recruited, contacted or interacted with, and no private data were accessed. Public accessibility was not treated as unrestricted ethical licence, because online material can retain privacy, contextual, and traceability risks (48). Usernames and direct URLs were removed and working identifiers were pseudonymised. All participant material in this article is reported as close paraphrase attributed to an internal thread identifier, and no contributor wording is reproduced verbatim, because even short quoted strings from Reddit can be traced to their source through search engines. To assess trace-back risk, draft extracts were searched against Reddit and a general web search on 2 August 2026, and every account was then rewritten as paraphrase so that no contributor wording remains to be matched. Some contributors may be located in the United Kingdom or the European Union, and their public posts were handled as personal data processed for research, with data minimisation, pseudonymisation, and restricted storage of the working corpus. Subreddit membership and Reddit scores were not treated as evidence of professional identity, truth, or representativeness.

### Use of generative artificial intelligence in manuscript preparation

To assist with proofreading and language refinement, the authors utilised Gemini integrated within Google Docs (Google) alongside Grammarly (Grammarly Inc.). No Reddit source extracts, comment-level data, or screening material were supplied to these services. The underlying model versions were not displayed by the service providers at the time of access. Generative artificial intelligence was not employed in the capacity of an author, data source, participant, coder, or independent analyst. The authors manually verified all edited text against the original study materials and cited literature, retaining full responsibility for the integrity of the manuscript. The authors reviewed, verified, and take responsibility for all revised content.

## Results

The retained corpus comprised 64 threads with 2,848 comments and replies from 1,723 distinct pseudonymised accounts, and 49 threads contributed to the final themes. Professional identities were not inferred from community membership and occupational roles were used only where contributors explicitly self-positioned. The four themes in the following are qualitative patterns rather than prevalence estimates, and Themes 3 and 4 are presented as propositions whose evidential base is narrower than that of Themes 1 and 2. Participant material is given as paraphrase followed by an internal thread identifier, evidence stream, and community, which link each account to the inventory in Supplementary Table S4. Table 5 reports anchor, supporting, boundary, and adjacent threads for each theme relative to the relevant evidence stream.

**Table 5:** Theme dispersion relative to the relevant evidence streams.

Final theme	Anchor threads (supporting, boundary, adjacent)	Evidence stream of anchors	Communities of anchor threads	Interpretation
1. Discharge readiness functions as a distributed longitudinal process rather than a static documentary state	12 (14, 1, 6)	11 discharge stream, 1 AI stream	r/nursing 7, r/NICU 2, r/medicine 2, r/pediatrics 1	Neonatal and maternal–newborn pattern. Two general-discharge anchors (T43, T54) are read as comparators. Lower-bound count
2. The structural decoupling of frontline clinical responsibility from institutional decision-making authority	5 (15, 5, 3)	5 discharge stream	r/nursing 3, r/medicine 2	Cross-cutting pattern. Five boundary threads (T17, T28, T40, T46, T49) describe workable distributed oversight
3. Accounts of residual verification and reconstruction work after AI-assisted drafting	4 (0, 0, 4)	4 of the 7 AI stream threads	r/medicine 3, r/healthIT 1	Direct AI pattern. Loss of rationale rests mainly on T7 and is reported narrowly
4. Perceived risks when anticipated efficiency gains are translated into higher workload	7 (5, 0, 5)	7 discharge stream	r/nursing 6, r/medicine 1	Proposition. AI-linked material in supporting threads T4 and T62 is predictive rather than first-hand

Table 5 counts are drawn from the thread-to-theme matrix. Anchor threads carried a theme's central interpretation, while supporting, boundary, and adjacent threads extended, limited, or contextualised it. The counts demonstrate dispersion across conversations, not prevalence. Theme 3 has four anchor threads, all within the seven-thread AI stream, and three adjacent AI stream threads. Theme 4 is different in kind. Its anchor threads are workload discussions from the discharge stream, and its AI-linked material consists of predictions and complaints in two supporting threads rather than a first-hand account of AI-driven workload change, so the theme is written as a proposition.

### Theme 1: Discharge readiness functions as a distributed longitudinal process rather than a static documentary state

Across the discharge stream threads, discharge readiness was constructed as a longitudinal process built across multiple relationships, rather than a static state captured within a final documentary artefact. Participants’ accounts suggested that because clinical judgements are distributed across various practitioners, assessments, and time points, access to a completed health record did not guarantee access to the situated, contextual knowledge they described as necessary to evaluate a patient's readiness to transition home.

Neonatal and postpartum discussions constructed readiness as something produced across time. In a thread opened by a nurse about to become a unit's first NICU case manager (T9, discharge stream, r/nursing), the poster described the existing process as disorganised and asked how other units managed it. Replies moved attention away from the final day. One contributor described a unit in which pending discharge needs were tracked visibly at the bedside as they arose, and another described a checklist given to parents at admission, with car seat and feeding milestones worked through over the stay. Readiness in these accounts was accumulated rather than declared.

A case management thread, concerned mainly with adult acute care and read here as a general comparator rather than neonatal evidence (T54, discharge stream, r/nursing), made the distribution of discharge knowledge explicit. One case manager described planning for discharge from the day of admission and gathering what was needed through bedside assessment, conversations with nurses, families, therapists and physicians, and chart review. The chart was one source within a wider network. Responsibility for synthesising these inputs could nevertheless become concentrated. The same contributor described being expected to produce a plan quickly while different specialties disagreed about whether a patient was ready, so that the role depended on many other people while carrying the expectation of a fast and safe discharge.

Postpartum and mother–baby discussions extended this pattern to caregiver capability. In one thread (T53, discharge stream, r/nursing), a mother–baby nurse described teaching a parent to become self-sufficient before going home, because the nurse would not be present once mother and baby left, and replies defined the nursing role as education rather than doing tasks for the family. Elsewhere, discharge education was described as a central part of postpartum work (T13), advice to a nurse moving into mother–baby care stressed understanding the conditions that delay discharge so that families could be helped to accept a longer stay (T11), and a postpartum nurse described explaining newborn temperature regulation to parents several times without persuading them (T22). Readiness here depended on what families had learnt, and that learning was produced in conversation over the stay rather than recorded in one document. An AI stream maternity discussion provided a complementary case, in which a labour-and-delivery nurse described parents arriving with limited preparation for newborn care and consulting ChatGPT during care, adding educational and interpretive work before transition home (T63, AI stream, r/nursing).

NICU discussions also represented readiness as provisional. After a parent described changing discharge estimates (T17, discharge stream, r/NICU), a self-described NICU nurse replied that units prefer caution because an infant who cannot yet regulate temperature may gain weight poorly and be readmitted. Another parent in the same thread described a physician proposing removal of a feeding tube before nurses argued successfully for one more day and an earlier estimate that changed once the physician registered that the infant was preterm rather than simply large. A further NICU account, an initiating post without replies (T58, discharge stream, r/nursing), described an infant approaching medical discharge criteria with no safe home to go to, so that clinical readiness and actual transition diverged.

Readiness also extended past discharge and across organisations. A parent whose infant had spent a month in the NICU described doubting, once home, whether the baby was feeding enough despite normal nappies, and a self-described NICU nurse replied by suggesting weights at home and asking what the paediatrician had found (T36, discharge stream, r/NICU). A family physician new to newborn care asked whether the practice should retrieve newborn screening results itself. Replies disagreed about whether the state programme, the inpatient physician who ordered the test, or the community practice held that duty, and one contributor advised checking every result at the 2-week review (T20, discharge stream, r/medicine). In a discussion of a newborn with a positive antibody test, a hospitalist described nursing pressure to replace serum bilirubin checks with transcutaneous readings before discharge, and replies disagreed about which protocol should apply (T30, discharge stream, r/pediatrics).

Across these accounts, the capacity to judge discharge depended upon information generated through different actors and at different points in the clinical trajectory. Discharge knowledge was therefore constructed as distributed and longitudinal. This directly addresses the verification capacity component of the research question. Access to a final record did not necessarily equate to access to all of the situated knowledge through which readiness had been established.

### Theme 2: The structural decoupling of frontline clinical responsibility from institutional decision-making authority

Participants’ accounts constructed a structural separation between the duty to recognise a clinical problem and the institutional power to prevent it. Across these discussions, healthcare professionals described bearing responsibility to educate, document, and escalate unsafe scenarios while remaining dependent upon external hierarchies to finalise decisions, a pattern that, in these accounts, left frontline patient advocacy vulnerable to administrative override.

This pattern appeared where participants constructed responsibility through duties to educate, document, challenge, refuse endorsement or escalate, but found that these duties did not necessarily confer control. A NICU discharge discussion provided the clearest instance (T39, discharge stream, r/nursing). The poster described a discharge that nurses and physicians considered unsafe, which went ahead after another department approved it, with the nurse manager completing the paperwork once every nurse had declined to sign, and closed by asking how such outcomes could be prevented. Nurses were positioned as able to recognise and document a risk and to withhold endorsement without being able to stop the decision. Replies complicated the account by questioning whether social work actually held the authority attributed to it, and one contributor stated that in their experience social workers do not clear families for discharge. That reply changed our reading of the case. It moved the analytic question from whether nurses lacked authority to whether any consequential route existed at all, since the actor to whom authority had been attributed may not have held it either.

A paediatric nurse practitioner described a contrasting arrangement (T40, discharge stream, r/pediatrics, initiating post without replies). The poster was sometimes the only clinician to see a boarding patient on a given day, yet any discharge required an attending review immediately before the patient left. Assessment and final discharge authority were located differently, but the separation was built into the model and an escalation route was always available. We read this as distributed oversight rather than asymmetry. Set beside T39, the contrast marks the boundary of the concept. In T39, a recognised risk had no route to becoming consequential, whereas in T40, a route was guaranteed and the poster's concern was limited support for learning rather than an inability to act.

The same separation appeared in physicians’ and nurses’ accounts elsewhere. In an adult discharge thread, an internal medicine resident described not feeling safe discharging about half the patients on the list while having little say as a resident (T43, discharge stream, r/medicine). A NICU discussion of perinatal HIV described staff who knew an infant needed treatment while the mother asked that her status be withheld from the infant's father, and a paediatric infectious diseases fellow described this as one of the most ethically unclear parts of the work, where teams begin by encouraging mothers to disclose (T23, discharge stream, r/medicine). A postpartum nurse described parents disbelieving explanations that they accepted once a physician repeated them, and a reply joked about leaving the room and returning in a white coat to be believed (T22, discharge stream, r/nursing). In each account, knowledge did not carry the authority to make it count.

Responsibility was also negotiated through escalation. In a postpartum discussion about nurses reportedly advising parents against newborn vaccination (T41, discharge stream, r/nursing), several replies urged the poster to report the behaviour to management or the state board of nursing. When the poster said they had already told the physician, a reply judged that insufficient. The exchange constructed professional responsibility as requiring access to a route through which a concern could become consequential rather than merely recognising or voicing it.

Other branches of the same thread set limits to that responsibility. One contributor proposed documenting a refusal and informing the prescriber, and another argued that parents keep the right to refuse and that supporting that right is part of nursing work. When a participant invoked the infant's interests, a reply drew a sharper boundary, stating that the nurse was not the child's parent and that decisions for other people's children lay outside the nursing role. Participants distinguished advocacy and education from decision-making authority.

Patient and family use of AI prompted related boundary work, although the evidence here is adjacent and limited. A labour-and-delivery nurse described parents arriving with little preparation for newborn care and consulting ChatGPT about their care in front of the nurse, just after the nurse had invited their questions (T63, AI stream, r/nursing). In a thread about hostile public posts written by patients’ relatives, one reply observed that people now consult ChatGPT and expect clinicians to follow what it says (T6, AI stream, r/nursing). In both, AI was used by families rather than clinicians. Its effect was to add explanation and correction to nursing work while leaving the nurse answerable for professional advice.

Across these discussions, responsibility and authority were constructed as related but non-equivalent. Participants could possess relevant knowledge and duties to act while remaining dependent upon another professional or organisational mechanism for consequential action. The authority component of the research question was therefore not reducible to formal hierarchy. It concerned whether recognised problems could actually alter an outcome.

### Theme 3: Accounts of residual verification and reconstruction work after AI-assisted drafting

In these accounts, AI did not remove documentation work. Participants described it as moving effort from composition towards checking and editing. In a narrower set of accounts, centred on one discharge summary discussion, generated text was said to omit the clinical rationale behind treatment decisions, so that its usefulness depended on a reviewer who could recognise the omission and recover the reasoning from source records.

AI-specific discussions constructed verification as residual professional work that remained after automated drafting. Participants did not divide neatly into those who trusted or rejected AI. Instead, they negotiated whether reductions in composition work justified the subsequent burden of checking, correcting, and reconstructing clinical meaning.

A clinician working in inpatient rehabilitation opened one discussion after receiving an AI-generated discharge summary from a referring hospital (T7, AI stream, r/medicine). The summary was described as well written but uninformative. It listed the medications started without explaining why an unusual analgesic combination or an anticonvulsant dose had been chosen, whether a seizure had occurred, or whether any plan existed to reduce the dose. The poster noted that the medication record already showed what had been given. What was missing was the reasoning. A reply described the same problem as prose that never said why a treatment had been chosen. The problem was not fabrication. A summary could reproduce clinical events while omitting the relationships and reasoning needed to interpret them.

A contributor to the same thread who had used self-hosted models for discharge summaries described the resulting responsibility directly. The models made mistakes and tended to strip out detail, so the clinician had to read, change, and restore it, and still found the tool time-saving overall. Another reply accepted AI discharge summaries provided they were proofread, while noting that a generated summary can be no better than the progress notes it draws on (T7). Verification was not in itself evidence that the system lacked value. The question was whether residual checking remained proportionate to the work removed.

Proportionality was debated elsewhere. A physician who had tried AI scribes described giving up on them because every note still had to be reviewed and edited, and because writing a note was itself the moment at which clinical thinking was organised and checked (T3, AI stream, r/medicine). In a thread on prompts for prior-authorisation letters (T2, AI stream, r/healthIT), the poster's own prompt instructed the model to flag missing information rather than fill gaps with assumptions, and replies warned that generated letters could invent facts. Prior knowledge was constructed as part of verification capacity. AI output was easier to scrutinise where users already knew enough to recognise implausibility or omission.

A large discussion of an electronic record summarisation feature produced divergent assessments (T62, AI stream, r/medicine). The initiating clinician described the summaries as often wrong and objected to the use of clinicians’ notes to produce them. Others regarded the output as a useful orientation to a patient, provided it was checked and edited. Source traceability changed the judgement for several participants, who noted that each statement in the summary linked to the note it drew on and described those links as what made the tool useful. A self-described paediatric anaesthetist, who often had only minutes to review long records for complex children before urgent cases, treated the summary as a starting point for chart review rather than a substitute for it.

A counter-case in the same thread showed that substantive review could coexist with meaningful time savings. One clinician described always reading the generated summary, checking consultation details and adding what was missing, and estimated that the tool had at least halved the time spent and left more time for ward work (T62).

Adjacent threads extended this logic beyond documentation tools, and they are reported as context rather than as evidence of verifying generated records. The physician in T3 also described scripted AI workflows that degraded as the underlying model changed, so that outputs had to be rechecked periodically. In a paediatric primary-care thread, the only AI mentioned was an ambient scribe whose roll-out had been paused, valued as relief from note-writing rather than for any checked output (T4, AI stream, r/pediatrics). In the governance thread, prompted by a proposal to prioritise low-cost AI avatars over frontline clinicians, a contributor who welcomed automation still described consistent quality as unsolved (T8, governance stream, r/medicine). Where patients or relatives brought ChatGPT advice into care, nurses described answering for and correcting it (T6, T63).

AI therefore shifted rather than eliminated documentation work. The direct AI evidence constructed verification capacity as dependent upon clinical knowledge, source accessibility, and the amount of reconstruction required. Verification demand increased where fluent output obscured missing reasoning or required extensive return to underlying records, but remained manageable where provenance was accessible and correction was bounded.

### Theme 4: Perceived risks when anticipated efficiency gains are translated into higher workload

This theme is written as a proposition. Its anchor threads are workload discussions from the discharge stream, and its AI-linked material consists of predictions and complaints in two supporting threads. Taken together, these accounts suggest that converting anticipated time savings into larger workloads may reduce the temporal capacity that verification requires. The mechanism is the contribution. Its prevalence has not been shown.

The AI-linked evidence came mainly from the summarisation thread (T62, AI stream, r/medicine). One participant predicted that administrators would meet any time saved with a larger case volume, since additional production was the only way to justify the spending. Another replied that clinicians would still have to check everything the tool produced, while managers would now argue that they should work faster because the AI had done the work. A third said they would rather see more patients than spend time on inefficient chart review. The participants were evaluating different levels of efficiency. One concerned the time taken by a particular documentation task. The other concerned the clinical capacity remaining after verification and workload reallocation.

A paediatric primary care thread showed how such expectations enter already strained work (T4, AI stream, r/pediatrics). The poster described exhaustion from same-day demands and from newborns discharged early without a follow-up plan, and presented an ambient scribe as the one hoped-for relief, while noting that the institution had paused its roll-out. The account is prospective. It shows the hope attached to automation where discharge follow-up already falls on stretched clinicians, not what happens once savings are realised.

The discharge stream workload accounts supplied the temporal side of the mechanism without reference to AI. A medical-surgical nurse with seven patients described being unable to complete the work and spending days off rehearsing what had been missed (T55, discharge stream, r/nursing). A labour-and-delivery nurse in orientation described one nurse carrying out tasks that other units spread across several roles, leaving little time to learn or debrief (T60, initiating post without replies). Removal of evening phlebotomy support moved blood sampling, including newborn screening before early discharge, onto nurses who had no backup when busy (T48, discharge stream, r/nursing). In an adult discharge thread, physicians linked premature discharge to reimbursement incentives, bed pressure, and burnout (T43, discharge stream, r/medicine).

Other workload anchors described the same pressure on time from different directions. A nurse caring for children described precepting students during the pandemic with patients sicker than the assignment assumed, and catching a wrongly charted temperature only by rechecking it personally (T10, discharge stream, r/nursing). A nurse floated to a newborn nursery described more mother–baby couplets than rooms and staff rushing to discharge as fast as possible (T15, discharge stream, r/nursing). A NICU technician described mothers being discharged before their babies to free beds during a labour and delivery diversion (T25, discharge stream, r/nursing). A medical-surgical nurse ready to leave the profession listed constant additions to charting requirements and repeated instructions to do more with less (T21, discharge stream, r/nursing).

Replies to the medical-surgical nurse sharpened the distinction between documentation and capacity. Contributors told the poster that the expectation was impossible, and one suggested that colleagues who appeared to cope were sometimes skilled mainly at getting things charted, which another reply described as cutting corners at the patient's expense (T55). Completed documentation was not treated as evidence that adequate time existed.

Across these accounts, efficiency was treated as contingent upon what happened to the time apparently saved. Where assistance removed bounded work and the saved time stayed with the clinician, as in the account of halved summary review (T62), participants described increased practical capacity. Where savings were expected to fund larger workloads while verification continued, participants anticipated a loss of the time that meaningful oversight needs. The evidence for this second pathway is predictive and indirect, and it should be tested prospectively.

## Discussion

The findings answer the research question by showing that responsibility, verification capacity, and authority were organisationally separable. Discharge stream evidence represented relevant knowledge as distributed across bedside observation, family learning, multidisciplinary coordination, and records, whereas direct artificial intelligence evidence retained professional responsibility for the final clinical representation after drafting was automated. Authority was also uneven because recognising, documenting, or escalating a concern did not necessarily confer control over the final decision or recurrent workflow. Oversight asymmetry is therefore a relational condition in which assigned responsibility and verification demand become disproportionate to the epistemic, temporal or institutional capacities available to the responsible actor.

Meaningful Human Control provides a broader normative standard that responsibility should remain commensurate with control capacity (2, 25). Healthcare artificial intelligence governance research similarly identifies epistemic capacity, cognitive space, decisional authority, and intervention effectiveness as conditions for meaningful oversight, while warning that clinician-in-the-loop arrangements can individualise responsibility without corresponding control (3, 23, 24). Oversight asymmetry adds a narrower organisational diagnosis, the relationship between a concrete verification obligation and the epistemic, temporal, and authority capacities available at that point. This directs organisations to ask who must verify, what substantive checking requires, and whether discrepancies can be made consequential. A nominal human checkpoint may therefore remain while practically exercisable oversight is absent. Table 1 summarises how this relational diagnosis differs from neighbouring accounts in unit of analysis, timing, and governance response.

Oversight asymmetry is also distinct from a conventional responsibility gap because responsibility need not be missing or indeterminate. Responsibility gap scholarship addresses difficulties in attributing culpability, moral accountability, public accountability, or active responsibility under distributed human–machine agency (28). In the present material, responsibility was often explicit. The clinician receiving a generated discharge summary remained answerable for continuing treatment, clinicians described themselves as responsible for checking generated text, and neonatal staff described duties to recognise or escalate unsafe discharge. The diagnostic problem is that answerability can exceed the capacities available to discharge it.

This prospective emphasis also complements moral crumple zone and liability sink accounts, which draw attention to the concentration of responsibility or liability around human operators despite limited control within wider sociotechnical systems (26, 27). Work on accountability and safety in healthcare AI, and on the liability exposure of clinicians who use generative tools, points to the same concentration of risk at the point of use (49, 50). Recent work on non-certified generative AI similarly shows that apparently low-friction clinical use can enter a regulatory and accountability grey zone once general-purpose systems begin to shape documentation or decision-making, which strengthens the case for assessing prospectively whether assigned responsibility is matched by workable verification conditions (51). Oversight asymmetry identifies a possible antecedent condition. A clinician may be positioned as the final reviewer while staffing, system design, workload expectations, and deployment decisions remain elsewhere. If an error subsequently escapes detection, the visible failure may be located at the human checkpoint even though the capacity to perform that checking was organisationally constrained. The concept therefore shifts attention from retrospective blame towards prospective proportionality between responsibility and practical control.

The relationship between Themes 1 and 3 is particularly important for understanding verification capacity within person-centred fundamental care. Neonatal discharge literature characterises readiness as longitudinal, multidisciplinary, and family-centred, involving medical history, treatment planning, caregiver competence, communication, and continuing education (29–34). The present findings connect this distributed epistemic environment to the direct AI discussions, where participants in one discharge summary discussion described summaries that were fluent yet omitted clinical reasoning. A worked example makes the cross-stream step visible. In T17, the decision to keep a feeding tube for another day rested on nurses’ bedside observation of feeding, and in T7, a clinician receiving a generated summary could see what had been given but not why. If a generated neonatal discharge summary was drawn from notes that did not capture that bedside reasoning, its reviewer would face the T7 problem in the T17 setting, and verification would depend on reaching the nurses and parents who held the missing knowledge. This extends verification complexity theory by suggesting that generative documentation may require not only error detection but also reconstructive verification, in which a reviewer returns to source records to recover reasoning, chronology, or context (16). The reviewer may therefore need to reconstruct why events occurred, how judgements changed, and which contextual relationships were lost during summarisation.

From a person-centred fundamental care perspective, the concern is therefore broader than documentary accuracy. A fluent summary may remain clinically inadequate if it fails to preserve the relational, educational, or contextual knowledge through which an individual's readiness, needs, and family capability have been understood. This interpretation is consistent with work defining fundamental care through integrated relational, psychosocial, and physical dimensions (35, 36).

Fluent output may also lower the apparent cost of scrutiny. Automation bias research shows that incorrect automated advice can influence professional judgement (17, 18), while explainability does not consistently calibrate trust (19). Reviews of clinical artificial intelligence implementation identify data access, workload, time, professional autonomy, understanding, and organisational readiness as conditions of effective use (20–22). Healthcare oversight analyses likewise conclude that clinician presence is insufficient when informed questioning and intervention are not practically possible (3, 24). Simply instructing clinicians to “check the artificial intelligence” may therefore be inadequate where checking depends upon dispersed knowledge, reconstructive work, or constrained authority.

Ambient scribe studies report lower documentation burden alongside continuing concerns about review, editing, accuracy, and completeness (10, 11, 52), and reviews of AI-assisted documentation describe efficiency benefits together with sparse real-world evidence and a continuing need for professional checking (13–15). Large language model (LLM)-assisted documentation is already being developed for nursing use, which makes the allocation of review responsibility an applied question (53, 54). The findings support a distinction between production efficiency and verification efficiency.

Theme 4 suggests why temporal capacity may be organisational rather than purely individual. Participants did not reject efficiency itself. Generated summaries were described positively where they removed bounded work and left the saved time with the clinician. Concern arose where projected savings were expected to become additional capacity before residual work had been removed. Clinical documentation studies show that AI can reduce time spent on particular tasks, while workflow effects, editing requirements, and realised benefits vary across settings (10, 11, 15). Those studies do not determine how organisations reallocate saved time. The theme adds a governance proposition that time reclaimed through higher caseloads cannot also serve as protected capacity for substantive verification.

The findings also refine institutional authority. The conceptual framework distinguishes output-level authority, the capacity to amend or reject an individual artefact, from system-level authority over recurrent defects, workflow rules, and continued deployment. Theme 2 suggests that this distinction extends beyond AI because participants could recognise and escalate perceived discharge problems while lacking final decision-making authority. In AI-assisted workflows, the same pattern may allow a clinician to correct individual outputs while having little influence over the system or workload arrangement that repeatedly generates verification demands. Nursing and implementation reviews identify accountability, professional autonomy, organisational readiness, and governance as recurring conditions of AI adoption and use (20, 22). Recent healthcare oversight analyses further distinguish a clinician's ability to edit or override an output from institutional capacity to intervene in system operation or governance (3, 24). The existence of an edit function should therefore not be treated as equivalent to institutional control. Algorithmovigilance offers one way of making system-level authority concrete. Balendran and colleagues propose adapting pharmacovigilance practice, including case reporting, standardised terminology, causality assessment, and dissemination of known incidents, to AI systems in routine use (55). A complementary minimum-viable governance protocol proposed by Sirago and colleagues places incident reporting, accountability, and the prevention of algorithmic harm within a continuous clinical AI governance process (56). Applied to documentation, these approaches would specify who receives a clinician's report of a recurrent defect, who is obliged to respond, and how repeated reports change workflow rules, staffing assumptions, monitoring, or deployment. Table 6 includes these elements.

**Table 6:** Governance priorities and prospective evaluation for responsible digital paediatrics.

Governance priority	Organisational implication	Prospective evaluation focus
Verification demand and protected review capacity	Assess residual review, correction, and source-checking work rather than drafting time alone. Projected AI time savings should not automatically justify higher caseloads or reduced staffing before verification demand is measured (10, 11, 13, 15)	Review time; correction burden; conversion of automation savings into workload expectations
Provenance and source access	Source links and provenance may reduce retrieval burden, but they do not substitute for clinical expertise, time, or authority. Systems should make source material readily traceable where substantive verification is expected (19, 21)	Provenance access; source retrieval burden; need for reconstructive verification
Output-level authority	Reviewers require practical authority to amend, reject, or delay individual AI-generated outputs when discrepancies are identified. Nominal human review is insufficient where detected problems cannot alter the output or decision	Capacity to amend or reject outputs; unresolved discrepancies; time available to act
System-level authority and escalation	Distinguish authority over a single note from authority over recurrent defects, verification rules, staffing assumptions, and continued deployment. Meaningful oversight requires effective escalation and intervention routes (3, 23, 24). Escalated concerns need a named recipient with a duty to respond, and repeated reports should feed a monitoring process that can change workflow rules, staffing assumptions, or deployment (55, 56)	Escalation response; recurrent defects; changes to workflow, staffing or deployment rules; named recipient and time to response; changes made after repeated reports

The comparative design therefore supports a theory of conditions rather than an estimate of incidence. Discharge stream discussions provide evidence about how discharge knowledge, responsibility, and authority are organised, whereas AI discussions directly evidence verification and AI-mediated communication. Table 4 makes the inferential step explicit, and the propositions that follow should be evaluated prospectively in deployed clinical systems rather than read as proof that neonatal AI workflows already exhibit oversight asymmetry.

The findings also support the narrower concept of contingent transparency. Participants valued linked sources where these reduced the effort of locating information, yet source visibility did not restore omitted reasoning or create time for review. Explanations can raise or lower clinician trust rather than acting as a uniform safeguard (19), and information supports oversight only where it is joined to epistemic capacity, decisional authority, and routes for intervention (3, 21). Provenance helps where the reviewer has the expertise to interpret it, the time to interrogate it, and the authority to act on what it shows.

Taken together, the findings suggest a prospective pathway from automation to oversight asymmetry. Automated drafting can reduce text production work while leaving verification responsibility intact, and verification demand may remain substantial where clinical rationale or contextual knowledge must be reconstructed. If anticipated time savings are then converted into higher throughput, temporal capacity for substantive review may fall further. In paediatric settings, the epistemic problem may be amplified when verification depends on longitudinal bedside, multidisciplinary, and family knowledge. Figure 1 summarises this mechanism and the organisational points at which governance can intervene.

**Figure 1:**
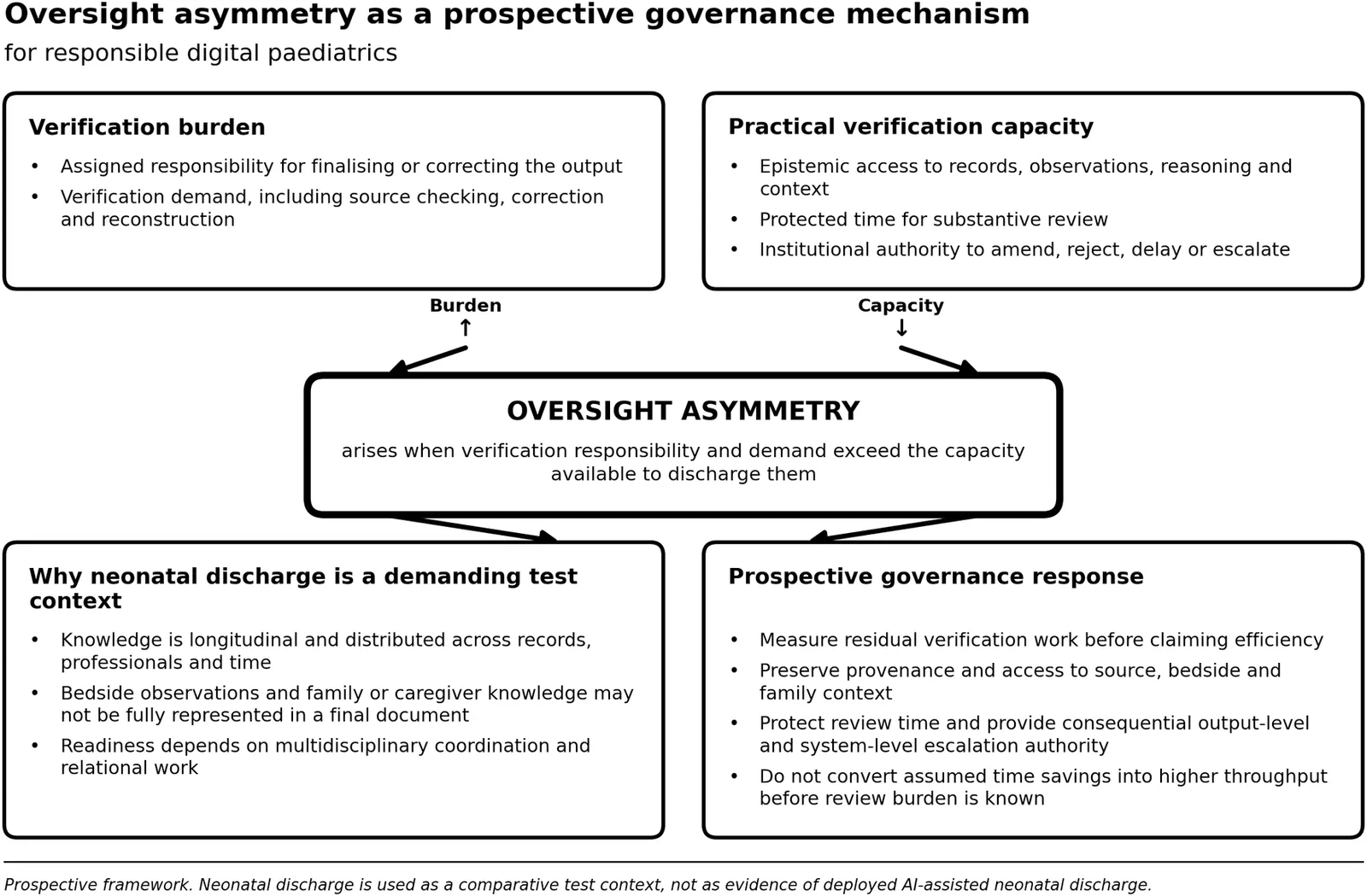
Oversight asymmetry as a prospective governance mechanism for responsible digital paediatrics. Oversight asymmetry may arise when assigned responsibility and verification demand exceed the epistemic, temporal, or institutional capacity available to the reviewer. Neonatal discharge is used as a comparative test context because relevant knowledge is longitudinal, multidisciplinary, and family-centred. The proposed governance response is to measure residual verification work, preserve provenance and access to source, bedside, and family context, protect review time, and provide consequential output-level and system-level escalation authority before anticipated efficiency is converted into higher throughput. The figure presents a prospective framework rather than evidence of deployed artificial intelligence-assisted neonatal discharge.

### Governance implications for responsible digital paediatrics

The governance implications are summarised in Table 6 and are framed for responsible digital paediatrics. The central requirement is to determine whether clinicians have sufficient information, time, and authority to verify artificial intelligence-generated documentation while preserving the relational, educational, and coordination work through which paediatric and family-centred care is delivered. A nominal human review step should not be treated as sufficient merely because a clinician remains formally responsible for the final output. These are prospective organisational propositions rather than demonstrated interventions, and they require evaluation in deployed paediatric workflows.

### Limitations

Several limitations constrain interpretation, as summarised in Table 7. The study analyses situated online discourse rather than directly observed clinical practice. Discharge stream material came predominantly from general nursing communities and included some adult discharge discussion, while the AI stream comprised seven threads, four of which anchored Theme 3. Reddit search matched terms loosely, which shaped the candidate pool, and an audit after screening identified 15 retained threads that should have been excluded and that contributed no evidence. The cross-stream analysis supports theory development and prospective propositions rather than estimates of incidence or causal effects. These propositions require testing in deployed clinical workflows.

**Table 7:** Study limitations and implications for interpretation.

Limitation	Implication for interpretation
Contributor identity and self-selection	Reddit identities, professional registration, and workplace roles could not be independently verified, and contributors self-selected into public discussion. Accounts should therefore be interpreted as situated discourse rather than verified descriptions of clinical practice
Purposive sampling and bounded retrieval	The corpus is neither exhaustive nor statistically representative. Platform search visibility, deleted or inaccessible material, and extraction ceilings may have influenced which discussions were retrievable, while unusual or contentious experiences may be over-represented
Search behaviour and screening consistency	Reddit search matched query terms loosely, and an audit after screening found 15 retained threads, mostly career-focused or general occupational discussions, that the stated criteria should have excluded. They supplied no evidence, but their retention shows that screening was less consistent than intended. Because the query set was built around neonatal discharge, the AI stream is unlikely to represent wider clinician discussion of AI documentation. Retrieval used the Data API before Reddit designated its research programme as the route for academic access
Community composition	General nursing and case management forums supplied most discharge stream material, while dedicated neonatal and NICU communities contributed a small minority of threads. Thirteen discharge stream threads concerned adult or unspecified settings, some retrieved through the term premature in the sense of early discharge. Findings should be read primarily as generalist nursing discourse touching on neonatal cases
Jurisdictional heterogeneity	Participants may have described different legal, staffing, and professional-authority arrangements, often without specifying location. Organisational interpretations cannot therefore be generalised to a single healthcare system or regulatory setting
Temporal change in AI systems	The 2021-to-2026 period spans substantial changes in generative AI products and clinical integration. Accounts concerning earlier systems should not be treated as descriptions of current technical performance
Evidence stream imbalance	The AI-specific corpus comprised seven documentation and communication threads plus one governance thread, substantially fewer than the neonatal stream. This supports comparative theory development but restricts claims about the breadth of AI practice
Online discourse rather than observed governance	The study examines how responsibility, knowledge, time, and authority are constructed in discussion, rather than directly observing staffing, review duration, institutional policy, or incident rates. Oversight asymmetry is therefore not estimated as an organisational incidence or causal effect
Abductive cross-stream inference	Neonatal discharge and AI-assisted documentation were compared across distinct evidence streams rather than observed together in one deployed workflow. The resulting propositions require prospective testing where AI-assisted documentation and high-stakes discharge work actually intersect

## Conclusion

Oversight asymmetry is a prospective governance diagnosis for situations in which assigned verification responsibility and verification demand exceed practical capacity. Discharge stream evidence suggests why responsible digital paediatrics cannot assume that clinically relevant knowledge is fully contained in a final documentary artefact, because readiness depends on longitudinal, multidisciplinary, and family knowledge. A small set of AI documentation discussions shows how automated drafting can leave professionals responsible for source checking, correction, and reconstruction after composition work is removed. Human oversight is meaningful only when verification demand is proportionate to epistemic access, practical time, and consequential authority. This study does not establish oversight asymmetry in deployed neonatal artificial intelligence systems or estimate its incidence. It instead identifies a mechanism that can be prospectively evaluated when artificial intelligence-assisted documentation is introduced into paediatric workflows, including measures of residual verification work, provenance access, protected review time, escalation effectiveness, and the organisational use of claimed efficiency gains.

## Data Availability

The data analysed in this study are subject to the following licenses/restrictions: The search protocol, query vocabulary, inclusion and exclusion criteria, and working codebook are reported in the Supplementary Material and are available from the corresponding author on reasonable request. The screening workbook, which lists Reddit submission identifiers and screening decisions without comment text, and the pseudonymised thread corpus are available to the editor and reviewers on request, so that any paraphrase can be checked against its source thread. The underlying comment-level dataset is not made publicly available because pseudonymisation does not remove re-identification risk for short, searchable text drawn from public accounts. Requests to access these datasets should be directed to atikus@aston.ac.uk.
