# Supplementary material for: Oversight asymmetry in AI-assisted clinical documentation: a comparative-abductive study using neonatal discharge as a test context
Source: Front Digit Health. 2026 Oct 28;8:1986499. doi: 10.3389/fdgth.2026.1986499 (PMC13752010; doi:10.3389/fdgth.2026.1986499)
Supplement: Supplementary file 1 [file Datasheet1.docx]

Supplementary Material

Oversight asymmetry in AI-assisted clinical documentation: a comparative-abductive study using neonatal discharge as a test context

This file replaces the supplementary file uploaded with the original submission, which was an internal audit information pack rather than the supporting material prepared for the article. It reports the search frame and configuration (S1), the full vocabulary and the exact 90 query strings (S2), the study flow (Figure S1), the eligibility criteria (S3), a thread-level inventory of the retained corpus (S4), the working codebook (S5), the relationship between this corpus and the authors’ previous Reddit studies (S6) and an updated SRQR map (S7). No contributor text is reproduced. Thread titles, stable pseudonymous identifiers and exact comment counts are withheld to limit trace-back, and the screening workbook with Reddit submission identifiers is available to the editor and reviewers on request.

# S1. Search frame and retrieval configuration

| Parameter | Specification |
| --- | --- |
| Target communities (16) | r/nursing, r/Nurses, r/Nurse, r/NursingUK, r/NursePractitioner, r/NICU_Nurses, r/NICU, r/neonatology, r/pediatrics, r/medicine, r/healthIT, r/HealthInformatics, r/AskDocs, r/Residency, r/medicalschool, r/healthcare |
| Communities returning candidate threads (8) | r/nursing, r/medicine, r/NICU, r/pediatrics, r/Nurses, r/NursingUK, r/healthIT, r/neonatology |
| Communities contributing retained threads (5) | r/nursing (45 threads), r/medicine (9), r/NICU (5), r/pediatrics (3), r/healthIT (2) |
| Query set | 90 strings generated by quota (Table S2b) |
| Search route | subreddit search within each target community, relevance sort, time filter all |
| Community-query routes | 1,440 (16 communities by 90 queries) |
| Submissions returned per route | Maximum 250 |
| Comments retrieved per thread | Maximum 900, replace_more() limit 3 |
| Deduplication | Reddit submission identifier used as the thread key across queries and communities |
| Posting window of retained threads | 2021 to 25 April 2026 (latest retained thread) |
| Data collection | Staged, between 3 March 2026 and 16 May 2026. Coding followed data collection |
| Software | Python with the Python Reddit API Wrapper (PRAW) |
| Platform access | Reddit Data API through a script application registered in October 2025. Existing access continued for non-commercial use when self-service access closed in November 2025. Reddit’s June 2026 policy update, published after data collection, now directs academic research through the Reddit for Researchers programme |
| Deleted accounts | Comments from deleted accounts retained their text without an account identifier, were pooled under one placeholder and excluded from counts of distinct accounts |
| Search engine behaviour | Reddit search appeared to match query terms loosely rather than requiring all terms, and the term premature also retrieved discussion of early adult discharge |

The script below records the community frame, the grouped vocabulary and the rule that generates the exact 90 query strings. Run as supplied, it returns 90 unique queries, 16 communities and 1,440 community-query routes. Retrieval credentials and local paths are not included.

"""

Reddit search protocol appendix script

Neonatal / AI documentation / discharge-transition home study

Purpose

-------

This script records the search ingredients requested for the Supporting Information:

1. The 16 target Reddit communities used for subreddit-level search routes.

2. The search vocabulary grouped by concept family.

3. The reproducible rule used to generate the exact 90 query combinations.

Terminology note

----------------

Outputs should be described as "retrieved public thread material", not as a

"full-thread corpus", because Reddit search/API access cannot prove that every

historical comment was retrieved.

Community-frame note

--------------------

The search frame lists 16 health-related Reddit communities spanning nursing,

neonatal/NICU, paediatrics, medicine, health informatics and wider

clinician/healthcare discussion.

"""

from __future__ import annotations

from pathlib import Path

import csv

\# -----------------------------------------------------------------------------

\# 1. Target Reddit communities / subreddit search routes

\# -----------------------------------------------------------------------------

TARGET_COMMUNITIES = [

\# Nursing communities

"nursing",

"Nurses",

"Nurse",

"NursingUK",

"NursePractitioner",

\# Neonatal / NICU / paediatric communities

"NICU_Nurses",

"NICU",

"neonatology",

"pediatrics",

\# Medicine and health-informatics communities

"medicine",

"healthIT",

"HealthInformatics",

\# Wider health-related communities

"AskDocs",

"Residency",

"medicalschool",

"healthcare",

]

SUBMISSIONS_PER_QUERY_LIMIT = 250

SEARCH_SORT = "relevance"

SEARCH_TIME_FILTER = "all"

SEARCH_ROUTE_TEMPLATE = (

"r/{community}.search(query, sort='relevance', time_filter='all', limit=250)"

)

\# -----------------------------------------------------------------------------

\# 2. Full query vocabulary / search terms grouped by concept family

\# -----------------------------------------------------------------------------

AI_RELATED_TERMS = [

"ai",

"artificial intelligence",

"chatgpt",

"gpt",

"llm",

"large language model",

"generative ai",

"machine learning",

"algorithm",

"ambient",

"automated",

"automation",

"dax",

"copilot",

"nuance",

"healthscribe",

"abridge",

"suki",

"deepscribe",

"nabla",

"augmedix",

"notable",

"heidi",

"freed",

]

DOCUMENTATION_COMMUNICATION_TERMS = [

"note",

"notes",

"chart",

"charting",

"documentation",

"document",

"scribe",

"dictation",

"transcription",

"summary",

"summaries",

"discharge summary",

"discharge summaries",

"discharge instructions",

"patient instructions",

"patient education",

"care plan",

"handover",

"hand-off",

"hand off",

"AI scribe",

"ambient scribe",

"ambient AI",

"ambient documentation",

"ambient clinical documentation",

"AI documentation",

"AI charting",

"AI note",

"AI notes",

"AI-generated note",

"AI generated note",

"automated documentation",

"automated note",

"digital scribe",

"medical scribe AI",

"clinical documentation AI",

"ChatGPT documentation",

"ChatGPT charting",

"ChatGPT notes",

"GPT documentation",

"LLM documentation",

"Copilot documentation",

]

NEONATAL_TERMS = [

"nicu",

"neonatal",

"neonate",

"neonates",

"neonatology",

"newborn",

"newborns",

"preterm",

"pre-term",

"premature",

"prematurity",

"scbu",

"special care baby",

"special care baby unit",

"special care nursery",

"neonatal intensive care",

"nursery",

"mother baby",

"postpartum",

"labour",

"labor and delivery",

"l&d",

"baby",

]

DISCHARGE_TRANSITION_HOME_TERMS = [

"discharge",

"discharged",

"discharging",

"discharge summary",

"discharge summaries",

"discharge instructions",

"discharge teaching",

"discharge education",

"transition home",

"going home",

"home",

"homegoing",

"home-going",

"home transition",

"parent education",

"family education",

"caregiver training",

"medication teaching",

"patient education",

"follow-up",

"follow up",

"after visit summary",

"avs",

]

\# -----------------------------------------------------------------------------

\# 3. Exact 90 systematically generated query combinations

\# -----------------------------------------------------------------------------

CORE_AI_DOCUMENTATION_SEEDS = [

"AI",

"ChatGPT",

"LLM",

"ambient scribe",

]

CORE_NEONATAL_CONTEXT_SEEDS = [

"NICU",

"neonatal",

"newborn",

"preterm",

]

CORE_DISCHARGE_EDUCATION_SEEDS = [

"discharge",

"discharge summary",

"discharge instructions",

]

DIRECT_AI_DOCUMENTATION_PHRASES = [

"AI scribe",

"ambient scribe",

"ambient AI",

"ambient documentation",

"ambient clinical documentation",

"AI documentation",

"AI charting",

"AI note",

"AI notes",

"AI-generated note",

"AI generated note",

"automated documentation",

"automated note",

"digital scribe",

"medical scribe AI",

"clinical documentation AI",

"discharge summary AI",

"discharge instructions AI",

]

VENDOR_PHRASES = [

"DAX Copilot",

"Nuance DAX",

"Dragon Ambient",

"HealthScribe",

"Abridge",

"Suki",

"DeepScribe",

"Nabla",

"Augmedix",

"Notable",

"Freed",

"Heidi",

]

GENERIC_AI_DOCUMENTATION_PHRASES = [

"ChatGPT documentation",

"ChatGPT charting",

"ChatGPT notes",

"ChatGPT discharge",

"GPT documentation",

"GPT discharge",

]

NEONATAL_AI_SHORT_PHRASES = [

"AI NICU",

"ChatGPT NICU",

"LLM NICU",

"ambient NICU",

"scribe NICU",

"AI neonatal",

]

def quote_if_needed(term: str) -> str:

"""Quote multi-word or hyphenated search terms to preserve phrase searching."""

term = term.strip()

return f'"{term}"' if " " in term or "-" in term else term

def deduplicate_preserve_order(items: list[str]) -> list[str]:

"""Deduplicate query strings case-insensitively while preserving order."""

seen: set[str] = set()

out: list[str] = []

for item in items:

clean = item.strip()

key = clean.lower()

if clean and key not in seen:

seen.add(key)

out.append(clean)

return out

def build_core_cartesian_queries() -> list[str]:

"""

Generate the core neonatal-discharge AI query block.

Rule: 4 AI/documentation seeds × 4 neonatal-context seeds × 3

discharge/education seeds = 48 core query strings.

"""

queries: list[str] = []

for ai in CORE_AI_DOCUMENTATION_SEEDS:

for clinical in CORE_NEONATAL_CONTEXT_SEEDS:

for discharge in CORE_DISCHARGE_EDUCATION_SEEDS:

queries.append(

f"{quote_if_needed(ai)} {quote_if_needed(clinical)} {quote_if_needed(discharge)}"

)

return queries

def exact_90_query_list() -> list[str]:

"""

Return the exact 90 query strings.

The 90 are generated by quota rather than by taking the first 90 from a

larger Cartesian list. This keeps vendor-specific and direct AI-documentation

searches inside the executed 90-query set.

Composition:

- 48 core Cartesian neonatal-discharge AI queries

- 18 direct AI-documentation phrases

- 12 vendor/product phrases

- 6 generic AI-documentation phrases

- 6 neonatal-AI short phrases

After case-insensitive deduplication, the list is asserted to contain exactly

90 unique query strings.

"""

queries = []

queries.extend(build_core_cartesian_queries())

queries.extend(DIRECT_AI_DOCUMENTATION_PHRASES)

queries.extend(VENDOR_PHRASES)

queries.extend(GENERIC_AI_DOCUMENTATION_PHRASES)

queries.extend(NEONATAL_AI_SHORT_PHRASES)

queries = deduplicate_preserve_order(queries)

assert len(queries) == 90, f"Expected 90 unique queries; found {len(queries)}"

assert any("DAX" in q or "HealthScribe" in q or "Abridge" in q for q in queries), (

"Vendor-specific queries must be present inside the exact 90-query set."

)

return queries

def community_query_routes() -> list[dict[str, str | int]]:

"""Return all subreddit-query routes implied by the search frame."""

routes: list[dict[str, str | int]] = []

for community in TARGET_COMMUNITIES:

for number, query in enumerate(exact_90_query_list(), start=1):

routes.append(

{

"community": community,

"query_no": number,

"query": query,

"route": SEARCH_ROUTE_TEMPLATE.format(community=community),

"submissions_per_query_limit": SUBMISSIONS_PER_QUERY_LIMIT,

"sort": SEARCH_SORT,

"time_filter": SEARCH_TIME_FILTER,

}

)

return routes

if __name__ == "__main__":

queries = exact_90_query_list()

routes = community_query_routes()

print(f"Target communities in the search frame: {len(TARGET_COMMUNITIES)}")

print(f"Exact query strings: {len(queries)}")

print(f"Community-query routes: {len(routes)}")

print(f"Submissions per query limit: {SUBMISSIONS_PER_QUERY_LIMIT}")

print("\nTarget communities:")

for community in TARGET_COMMUNITIES:

print(f"- {community}")

print("\nExact 90 queries:")

for number, query in enumerate(queries, start=1):

print(f"{number:02d}. {query}")

out = Path("Exact_90_Query_List_From_Script.csv")

with out.open("w", newline="", encoding="utf-8-sig") as f:

writer = csv.DictWriter(f, fieldnames=["query_no", "query"])

writer.writeheader()

for number, query in enumerate(queries, start=1):

writer.writerow({"query_no": number, "query": query})

# S2. Search vocabulary and exact query strings

## Table S2a. Search vocabulary by concept family

| Concept family | Terms |
| --- | --- |
| AI-related terms | ai, artificial intelligence, chatgpt, gpt, llm, large language model, generative ai, machine learning, algorithm, ambient, automated, automation, dax, copilot, nuance, healthscribe, abridge, suki, deepscribe, nabla, augmedix, notable, heidi, freed |
| Documentation and communication | note, notes, chart, charting, documentation, document, scribe, dictation, transcription, summary, summaries, discharge summary, discharge summaries, discharge instructions, patient instructions, patient education, care plan, handover, hand-off, hand off, AI scribe, ambient scribe, ambient AI, ambient documentation, ambient clinical documentation, AI documentation, AI charting, AI note, AI notes, AI-generated note, AI generated note, automated documentation, automated note, digital scribe, medical scribe AI, clinical documentation AI, ChatGPT documentation, ChatGPT charting, ChatGPT notes, GPT documentation, LLM documentation, Copilot documentation |
| Neonatal and maternal-newborn context | nicu, neonatal, neonate, neonates, neonatology, newborn, newborns, preterm, pre-term, premature, prematurity, scbu, special care baby, special care baby unit, special care nursery, neonatal intensive care, nursery, mother baby, postpartum, labour, labor and delivery, l&d, baby |
| Discharge and transition home | discharge, discharged, discharging, discharge summary, discharge summaries, discharge instructions, discharge teaching, discharge education, transition home, going home, home, homegoing, home-going, home transition, parent education, family education, caregiver training, medication teaching, patient education, follow-up, follow up, after visit summary, avs |

## Table S2b. The 90 query strings applied in each target community

| No. | Query string | Block |
| --- | --- | --- |
| 1 | AI NICU discharge | Core combination |
| 2 | AI NICU "discharge summary" | Core combination |
| 3 | AI NICU "discharge instructions" | Core combination |
| 4 | AI neonatal discharge | Core combination |
| 5 | AI neonatal "discharge summary" | Core combination |
| 6 | AI neonatal "discharge instructions" | Core combination |
| 7 | AI newborn discharge | Core combination |
| 8 | AI newborn "discharge summary" | Core combination |
| 9 | AI newborn "discharge instructions" | Core combination |
| 10 | AI preterm discharge | Core combination |
| 11 | AI preterm "discharge summary" | Core combination |
| 12 | AI preterm "discharge instructions" | Core combination |
| 13 | ChatGPT NICU discharge | Core combination |
| 14 | ChatGPT NICU "discharge summary" | Core combination |
| 15 | ChatGPT NICU "discharge instructions" | Core combination |
| 16 | ChatGPT neonatal discharge | Core combination |
| 17 | ChatGPT neonatal "discharge summary" | Core combination |
| 18 | ChatGPT neonatal "discharge instructions" | Core combination |
| 19 | ChatGPT newborn discharge | Core combination |
| 20 | ChatGPT newborn "discharge summary" | Core combination |
| 21 | ChatGPT newborn "discharge instructions" | Core combination |
| 22 | ChatGPT preterm discharge | Core combination |
| 23 | ChatGPT preterm "discharge summary" | Core combination |
| 24 | ChatGPT preterm "discharge instructions" | Core combination |
| 25 | LLM NICU discharge | Core combination |
| 26 | LLM NICU "discharge summary" | Core combination |
| 27 | LLM NICU "discharge instructions" | Core combination |
| 28 | LLM neonatal discharge | Core combination |
| 29 | LLM neonatal "discharge summary" | Core combination |
| 30 | LLM neonatal "discharge instructions" | Core combination |
| 31 | LLM newborn discharge | Core combination |
| 32 | LLM newborn "discharge summary" | Core combination |
| 33 | LLM newborn "discharge instructions" | Core combination |
| 34 | LLM preterm discharge | Core combination |
| 35 | LLM preterm "discharge summary" | Core combination |
| 36 | LLM preterm "discharge instructions" | Core combination |
| 37 | "ambient scribe" NICU discharge | Core combination |
| 38 | "ambient scribe" NICU "discharge summary" | Core combination |
| 39 | "ambient scribe" NICU "discharge instructions" | Core combination |
| 40 | "ambient scribe" neonatal discharge | Core combination |
| 41 | "ambient scribe" neonatal "discharge summary" | Core combination |
| 42 | "ambient scribe" neonatal "discharge instructions" | Core combination |
| 43 | "ambient scribe" newborn discharge | Core combination |
| 44 | "ambient scribe" newborn "discharge summary" | Core combination |
| 45 | "ambient scribe" newborn "discharge instructions" | Core combination |
| 46 | "ambient scribe" preterm discharge | Core combination |
| 47 | "ambient scribe" preterm "discharge summary" | Core combination |
| 48 | "ambient scribe" preterm "discharge instructions" | Core combination |
| 49 | AI scribe | Direct AI-documentation phrase |
| 50 | ambient scribe | Direct AI-documentation phrase |
| 51 | ambient AI | Direct AI-documentation phrase |
| 52 | ambient documentation | Direct AI-documentation phrase |
| 53 | ambient clinical documentation | Direct AI-documentation phrase |
| 54 | AI documentation | Direct AI-documentation phrase |
| 55 | AI charting | Direct AI-documentation phrase |
| 56 | AI note | Direct AI-documentation phrase |
| 57 | AI notes | Direct AI-documentation phrase |
| 58 | AI-generated note | Direct AI-documentation phrase |
| 59 | AI generated note | Direct AI-documentation phrase |
| 60 | automated documentation | Direct AI-documentation phrase |
| 61 | automated note | Direct AI-documentation phrase |
| 62 | digital scribe | Direct AI-documentation phrase |
| 63 | medical scribe AI | Direct AI-documentation phrase |
| 64 | clinical documentation AI | Direct AI-documentation phrase |
| 65 | discharge summary AI | Direct AI-documentation phrase |
| 66 | discharge instructions AI | Direct AI-documentation phrase |
| 67 | DAX Copilot | Vendor or product |
| 68 | Nuance DAX | Vendor or product |
| 69 | Dragon Ambient | Vendor or product |
| 70 | HealthScribe | Vendor or product |
| 71 | Abridge | Vendor or product |
| 72 | Suki | Vendor or product |
| 73 | DeepScribe | Vendor or product |
| 74 | Nabla | Vendor or product |
| 75 | Augmedix | Vendor or product |
| 76 | Notable | Vendor or product |
| 77 | Freed | Vendor or product |
| 78 | Heidi | Vendor or product |
| 79 | ChatGPT documentation | Generic AI-documentation phrase |
| 80 | ChatGPT charting | Generic AI-documentation phrase |
| 81 | ChatGPT notes | Generic AI-documentation phrase |
| 82 | ChatGPT discharge | Generic AI-documentation phrase |
| 83 | GPT documentation | Generic AI-documentation phrase |
| 84 | GPT discharge | Generic AI-documentation phrase |
| 85 | AI NICU | Neonatal AI short phrase |
| 86 | ChatGPT NICU | Neonatal AI short phrase |
| 87 | LLM NICU | Neonatal AI short phrase |
| 88 | ambient NICU | Neonatal AI short phrase |
| 89 | scribe NICU | Neonatal AI short phrase |
| 90 | AI neonatal | Neonatal AI short phrase |

# Figure S1. Study flow

Identification. 90 query strings applied in each of 16 communities (1,440 community-query routes), up to 250 submissions per route. Returns were deduplicated before screening.

↓

Deduplication by Reddit submission identifier. 97 candidate threads from 8 communities, containing 3,823 comments and replies from 2,280 commenting accounts.

↓

Thread-level screening against the criteria in S3. 33 threads excluded against the criteria in S3, on grounds of incidental reference to AI, use of AI in a non-artificial-intelligence sense, predominantly career-related discussion, no substantive link to documentation, communication, discharge or responsibility, or insufficient retrievable material. The initial strict rule requiring AI documentation, neonatal care and discharge within the same thread was replaced before full coding by the six-area eligibility rule reported in the Methods.

↓

Retained. 64 threads containing 2,848 comments and replies from 1,723 pseudonymised accounts.

↓

Stream allocation by substantive subject. Discharge and transition home 49 threads (2,119 comments). AI documentation and communication 7 threads (423). Healthcare AI governance 1 thread (47). Not allocated 7 threads (259).

↓

Post-screening audit. 49 contributory threads (2,473 comments). 15 non-contributory threads, 8 career-focused and 7 general occupational discussions, including 6 without retrievable replies. No extract or thematic evidence was drawn from non-contributory threads.

↓

Analysis. Within-stream reflexive thematic analysis, then abductive comparison through five sensitising dimensions, yielding four themes (manuscript Table 5).

# S3. Inclusion and exclusion criteria

| Inclusion criterion | Operational definition |
| --- | --- |
| AI-assisted clinical documentation or communication | Threads substantively discussing AI-generated or AI-assisted notes, summaries, charting, dictation, transcription, automated documentation, digital scribes, ambient documentation, or related clinical communication tools. |
| Named commercial or vendor AI documentation systems | Threads concerning the clinical use, implementation, accuracy, workflow consequences, professional responsibility, or governance of named systems or vendors, including DAX Copilot, Nuance DAX, Dragon Ambient, HealthScribe, Abridge, Suki, DeepScribe, Nabla, Augmedix, Notable, Freed, and Heidi. |
| Generative or algorithmic technologies used in healthcare documentation | Threads involving ChatGPT, GPT, large language models, generative AI, machine learning, algorithms, automation, or similar technologies where these were connected to healthcare documentation, communication, or clinical workflow. |
| AI-generated or AI-assisted discharge material | Threads discussing discharge summaries, discharge instructions, patient instructions, patient education, care plans, handovers, or other transition-related communication produced or assisted by AI. |
| Neonatal, NICU, newborn, preterm, postpartum, or maternal-newborn care | Threads in which discharge, transition home, readiness, follow-up, family education, caregiver preparation, continuity, or coordination of care was substantively discussed. |
| Neonatal or paediatric discharge and transition-home work | Threads addressing discharge teaching, discharge education, parent or family education, caregiver training, medication teaching, follow-up, after-visit information, or related transition activities. |
| Verification and correction of digitally generated clinical information | Threads describing checking generated text against source records, identifying errors or hallucinations, correcting omissions, reconstructing clinical reasoning, or determining whether generated content was adequate for clinical use. |
| Professional responsibility and accountability | Threads addressing who was expected to check, correct, explain, sign, approve, reject, delay, escalate, or otherwise answer for a clinical document, communication, or discharge decision. |
| Clinical authority and escalation | Threads examining whether professionals had practical authority to amend, reject, delay, escalate, or otherwise make a recognised concern consequential. |
| Workload, staffing, time pressure, or throughput | Threads where these materially affected documentation, verification, discharge preparation, communication, follow-up, or the practical capacity to provide clinical oversight. |
| Distribution of relevant clinical knowledge | Threads showing that information needed for safe discharge or verification was dispersed across records, professionals, bedside observations, patients, parents, families, or time. |
| Patient or family use of AI affecting clinical communication | Threads where AI-generated information altered the informational relationship between patients or families and clinicians or created additional explanation, correction, contextualisation, or verification work. |
| Healthcare AI governance or organisational implementation | Threads addressing deployment, accountability, staffing implications, professional autonomy, workflow redesign, risk management, procurement/implementation, or institutional decision-making concerning AI. |
| Sufficient retrievable conversational material | Threads with enough accessible content to permit contextual interpretation of the initiating post together with relevant top-level comments and nested replies. |

| Exclusion criterion | Operational definition |
| --- | --- |
| Incidental AI references | Threads in which AI was mentioned only incidentally and was not substantively connected to healthcare documentation, communication, discharge, clinical responsibility, or governance. |
| Non-artificial-intelligence uses of 'AI' | Threads where the term 'AI' referred to something other than artificial intelligence. |
| Predominantly career-related discussion | Job seeking, specialty choice, career change, training progression, employment security, or speculation about whether a profession was 'AI-proof' without substantive discussion of clinical workflow, documentation, verification, responsibility, or governance. |
| Vendor/product discussion without a clinical practice link | Threads focused primarily on a vendor, product, promotion, purchasing, or general technology discussion without meaningful connection to clinical use, verification, professional responsibility, workflow, patient communication, or governance. |
| General pro- or anti-AI opinion only | Threads consisting mainly of broad enthusiasm, scepticism, or speculation about AI without a situated healthcare documentation, communication, workflow, or governance issue. |
| General occupational stress or emotional burden only | Threads centred on burnout, grief, interpersonal conflict, or emotional strain without a substantive connection to documentation, discharge, verification, responsibility, authority, or relevant workload capacity. |
| Topical proximity without substantive relevance | Threads occurring in a relevant clinical community but not materially informing responsibility, verification capacity, authority, discharge readiness, digital-health practice, or governance. |
| Neonatal/newborn/postpartum material without relevant transition or oversight content | Threads mentioning neonatal, newborn, postpartum, or related settings but not substantively addressing discharge, transition, follow-up, education, coordination, responsibility, or another element relevant to the research question. |
| Single adverse event, disagreement, error, or workload complaint alone | These were not sufficient by themselves to establish oversight asymmetry. Evidence had to show a substantive relationship between assigned responsibility and the information, time, or authority available to discharge that responsibility. |
| Insufficient retrievable material | Threads where accessible content was too limited, inaccessible, or fragmented to support meaningful contextual interpretation. |
| Analytically non-contributory retained cases | Threads that remained in the audit material but did not materially contribute to final themes were treated as boundary, adjacent, or non-contributory cases rather than as direct thematic evidence. |

A post-screening audit found that 15 retained threads met the exclusion criteria for career-related discussion or general occupational experience. They are reported in S4 as non-contributory rather than removed, so that all counts remain traceable.

# S4. Thread-level inventory of the retained corpus

Stream is the evidence stream to which the thread was allocated by substantive subject. Setting is the clinical setting described in the thread. Theme roles are drawn from the thread-to-theme matrix, where an anchor carries a theme’s central interpretation, a supporting thread extends it, a boundary thread limits it and an adjacent thread supplies context. Posting years and Reddit submission identifiers are held in the screening workbook, which is available to the editor and reviewers on request.

| ID | Community | Stream | Setting | Retrieved comments | Contributory | Theme roles |
| --- | --- | --- | --- | --- | --- | --- |
| T1 | r/nursing | Not allocated | General or unspecified | More than 100 | No | None |
| T2 | r/healthIT | AI documentation and communication | General or unspecified | 1 to 10 | Yes | Theme 3 anchor |
| T3 | r/medicine | AI documentation and communication | General or unspecified | 51 to 100 | Yes | Theme 3 anchor |
| T4 | r/pediatrics | AI documentation and communication | Paediatric | 11 to 50 | Yes | Theme 1 supporting  Theme 2 supporting  Theme 3 adjacent  Theme 4 supporting |
| T5 | r/nursing | Not allocated | General or unspecified | 0 | No | None |
| T6 | r/nursing | AI documentation and communication | Maternal-newborn | 1 to 10 | Yes | Theme 1 adjacent  Theme 3 adjacent |
| T7 | r/medicine | AI documentation and communication | General or unspecified | 11 to 50 | Yes | Theme 3 anchor |
| T8 | r/medicine | Healthcare AI governance | General or unspecified | 11 to 50 | Yes | Theme 2 supporting  Theme 3 adjacent  Theme 4 adjacent |
| T9 | r/nursing | Discharge and transition home | Neonatal (NICU) | 1 to 10 | Yes | Theme 1 anchor |
| T10 | r/nursing | Discharge and transition home | Paediatric | 11 to 50 | Yes | Theme 1 supporting  Theme 4 anchor |
| T11 | r/nursing | Discharge and transition home | Maternal-newborn | 1 to 10 | Yes | Theme 1 supporting |
| T12 | r/nursing | Discharge and transition home | Maternal-newborn | More than 100 | Yes | Theme 2 adjacent |
| T13 | r/nursing | Discharge and transition home | Maternal-newborn | 11 to 50 | Yes | Theme 1 supporting  Theme 4 adjacent |
| T14 | r/nursing | Discharge and transition home | Maternal-newborn | 11 to 50 | Yes | Theme 1 adjacent |
| T15 | r/nursing | Discharge and transition home | General or unspecified | More than 100 | Yes | Theme 1 supporting  Theme 2 supporting  Theme 4 anchor |
| T16 | r/medicine | Discharge and transition home | General or unspecified | 11 to 50 | Yes | Theme 2 adjacent  Theme 4 adjacent |
| T17 | r/NICU | Discharge and transition home | Neonatal (NICU) | 1 to 10 | Yes | Theme 1 anchor  Theme 2 boundary |
| T18 | r/nursing | Discharge and transition home | Paediatric | 1 to 10 | No | None |
| T19 | r/nursing | Discharge and transition home | Neonatal (NICU) | 51 to 100 | Yes | Theme 1 adjacent |
| T20 | r/medicine | Discharge and transition home | Maternal-newborn | 11 to 50 | Yes | Theme 1 anchor  Theme 2 supporting |
| T21 | r/nursing | Discharge and transition home | General or unspecified | 11 to 50 | Yes | Theme 2 supporting  Theme 4 anchor |
| T22 | r/nursing | Discharge and transition home | Maternal-newborn | 11 to 50 | Yes | Theme 1 supporting  Theme 2 anchor |
| T23 | r/medicine | Discharge and transition home | Maternal-newborn | More than 100 | Yes | Theme 2 anchor |
| T24 | r/nursing | Discharge and transition home | General or unspecified | 1 to 10 | Yes | Theme 1 supporting  Theme 2 supporting |
| T25 | r/nursing | Discharge and transition home | Maternal-newborn | 11 to 50 | Yes | Theme 1 supporting  Theme 4 anchor |
| T26 | r/NICU | Discharge and transition home | Neonatal (NICU) | 1 to 10 | No | None |
| T27 | r/nursing | Discharge and transition home | General or unspecified | 11 to 50 | No | None |
| T28 | r/nursing | Discharge and transition home | Maternal-newborn | 1 to 10 | Yes | Theme 1 supporting  Theme 2 boundary |
| T29 | r/nursing | Discharge and transition home | General or unspecified | 1 to 10 | No | None |
| T30 | r/pediatrics | Discharge and transition home | Maternal-newborn | 1 to 10 | Yes | Theme 1 anchor  Theme 2 supporting |
| T31 | r/nursing | Discharge and transition home | General or unspecified | 1 to 10 | No | None |
| T32 | r/nursing | Discharge and transition home | General or unspecified | 1 to 10 | No | None |
| T33 | r/NICU | Discharge and transition home | Neonatal (NICU) | 11 to 50 | Yes | Theme 1 supporting |
| T34 | r/nursing | Discharge and transition home | Maternal-newborn | 51 to 100 | Yes | Theme 1 adjacent |
| T35 | r/NICU | Discharge and transition home | Neonatal (NICU) | 1 to 10 | Yes | Theme 1 supporting  Theme 2 supporting |
| T36 | r/NICU | Discharge and transition home | Neonatal (NICU) | 1 to 10 | Yes | Theme 1 anchor |
| T37 | r/nursing | Discharge and transition home | General or unspecified | 51 to 100 | No | None |
| T38 | r/nursing | Discharge and transition home | Maternal-newborn | 1 to 10 | No | None |
| T39 | r/nursing | Discharge and transition home | Neonatal (NICU) | 1 to 10 | Yes | Theme 1 anchor  Theme 2 anchor |
| T40 | r/pediatrics | Discharge and transition home | Paediatric | 0 | Yes | Theme 2 boundary  Theme 4 adjacent |
| T41 | r/nursing | Discharge and transition home | Maternal-newborn | 51 to 100 | Yes | Theme 1 supporting  Theme 2 anchor |
| T42 | r/nursing | Discharge and transition home | Paediatric | 1 to 10 | Yes | Theme 1 boundary |
| T43 | r/medicine | Discharge and transition home | General or unspecified | 11 to 50 | Yes | Theme 1 anchor  Theme 2 anchor  Theme 4 anchor |
| T44 | r/nursing | Discharge and transition home | Neonatal (NICU) | 1 to 10 | Yes | Theme 1 adjacent |
| T45 | r/nursing | Discharge and transition home | General or unspecified | 51 to 100 | Yes | Theme 2 adjacent |
| T46 | r/nursing | Discharge and transition home | Neonatal (NICU) | 11 to 50 | Yes | Theme 2 boundary |
| T47 | r/nursing | Discharge and transition home | Maternal-newborn | More than 100 | Yes | Theme 1 anchor  Theme 2 supporting |
| T48 | r/nursing | Discharge and transition home | Maternal-newborn | 1 to 10 | Yes | Theme 1 supporting  Theme 4 anchor |
| T49 | r/nursing | Discharge and transition home | Neonatal (NICU) | 11 to 50 | Yes | Theme 2 boundary |
| T50 | r/nursing | Discharge and transition home | Maternal-newborn | 51 to 100 | Yes | Theme 1 anchor  Theme 2 supporting |
| T51 | r/nursing | Discharge and transition home | Maternal-newborn | More than 100 | Yes | Theme 2 supporting  Theme 4 supporting |
| T52 | r/nursing | Discharge and transition home | Neonatal (NICU) | 1 to 10 | Yes | Theme 1 adjacent |
| T53 | r/nursing | Discharge and transition home | Maternal-newborn | 51 to 100 | Yes | Theme 1 supporting  Theme 2 supporting  Theme 4 supporting |
| T54 | r/nursing | Discharge and transition home | General or unspecified | 1 to 10 | Yes | Theme 1 anchor  Theme 2 supporting  Theme 4 adjacent |
| T55 | r/nursing | Discharge and transition home | General or unspecified | 11 to 50 | Yes | Theme 4 anchor |
| T56 | r/nursing | Not allocated | General or unspecified | 0 | No | None |
| T57 | r/nursing | Not allocated | General or unspecified | 0 | No | None |
| T58 | r/nursing | Discharge and transition home | Neonatal (NICU) | 0 | Yes | Theme 1 anchor |
| T59 | r/nursing | Not allocated | General or unspecified | 0 | No | None |
| T60 | r/nursing | Discharge and transition home | Maternal-newborn | 0 | Yes | Theme 2 supporting  Theme 4 anchor |
| T61 | r/healthIT | Not allocated | General or unspecified | 0 | No | None |
| T62 | r/medicine | AI documentation and communication | General or unspecified | More than 100 | Yes | Theme 2 supporting  Theme 3 anchor  Theme 4 supporting |
| T63 | r/nursing | AI documentation and communication | Maternal-newborn | More than 100 | Yes | Theme 1 anchor  Theme 3 adjacent |
| T64 | r/medicine | Not allocated | General or unspecified | 0 | No | None |

# S5. Working codebook

Codes remained open and revisable throughout the analysis and were not used to calculate agreement statistics. The negative-case rule and the sensitising dimensions were applied after provisional themes had been developed.

## Table S5a. First-cycle working codes

| Code | Operational definition | Include when | Exclude / distinguish from | Analytic indicators |
| --- | --- | --- | --- | --- |
| Assigning responsibility | Attribution of an obligation, duty or accountability to a person, role, team, organisation or system. | A contributor states or implies who is expected to check, decide, document, explain, follow up, escalate or ensure safety. | General descriptions of tasks where no responsibility or accountability is attributed. | Responsible for; expected to; must check; has to sign; should follow up; accountable for. |
| Refusing responsibility | Explicit rejection, resistance or disavowal of an assigned responsibility. | A contributor says a task, decision or liability should not belong to them or declines to accept it. | Simple inability to complete a task because of workload without an explicit responsibility claim. | Not my responsibility; refuse to sign; should not be on me; cannot accept liability. |
| Sharing responsibility | Construction of responsibility as distributed across multiple actors. | Care, discharge, documentation or follow-up is described as jointly held across professions, teams, families or organisations. | Sequential handoff where responsibility clearly transfers to one actor rather than remains shared. | Team responsibility; multidisciplinary; parents and staff; shared decision; multiple services. |
| Unclear ownership | Ambiguity about who is responsible for a task, decision, follow-up or consequence. | Contributors question who owns an action or describe gaps between roles or services. | Clear shared responsibility where roles are explicit. | Who is responsible?; falls between teams; nobody owns it; unclear who follows up. |
| Verification work | Active checking, comparison, validation or review required before information or a decision can be trusted or acted upon. | Clinicians compare generated or recorded information with source notes, observations, results, policy or clinical knowledge. | Routine reading without an evaluative checking function. | Double-check; cross-check; review; verify; compare with source; confirm accuracy. |
| Difficult-to-detect error | Error, omission or distortion that is plausible, subtle or not readily visible without contextual knowledge. | Discussion describes hallucinations, missing rationale, misleading summaries or errors that require expertise or source access to identify. | Obvious typographical mistakes or readily visible formatting issues. | Looks plausible but wrong; missing reasoning; subtle omission; confident but incorrect. |
| Access to source information | Availability of the underlying information needed to verify a claim, note, summary or decision. | A contributor refers to source notes, labs, bedside observations, prior records, family knowledge or links needed for checking. | General information access not connected to verification or decision-making. | Can see source note; trace back; underlying record; original result; provenance. |
| Information fragmentation | Relevant knowledge is dispersed across records, people, settings or time rather than available in one place. | The thread describes incomplete handoffs, multiple systems, longitudinal observations or knowledge split across actors. | Simple large volume of information without dispersion or access problems. | Scattered records; different teams know different things; not in one note; distributed knowledge. |
| Situated professional knowledge | Context-dependent clinical or professional knowledge developed through experience, role, familiarity or direct observation. | Contributors rely on bedside familiarity, clinical judgement, workflow knowledge or experiential understanding. | Formal guideline knowledge alone without situated or role-based interpretation. | I know this patient; bedside judgement; clinical experience; local workflow knowledge. |
| Caregiver knowledge | Information, observations or practical knowledge held by parents, families or carers that contributes to assessment or transition. | Family observations, learning, readiness, preferences or home context are treated as clinically relevant. | General patient satisfaction comments with no contribution to care knowledge. | Parent knows feeding pattern; caregiver teaching; family readiness; home context. |
| Time pressure | Insufficient or constrained time to complete care, documentation, review, verification or communication. | Contributors describe competing tasks, high caseload, rapid turnover, interruptions or lack of protected review time. | General dissatisfaction with work where time is not part of the problem. | No time; rushed; too many patients; competing demands; turnover pressure. |
| Verification as additional work | Checking or correcting automated/digital output creates residual work rather than eliminating effort. | AI-generated content must be proofread, reconstructed, corrected or compared with source material. | Verification that is negligible or clearly reduces overall workload without burden. | Still have to read everything; proofreading; correction burden; saved drafting but added checking. |
| Workarounds | Informal adaptations used to make a process function when formal systems, tools or workflows were inadequate. | A participant describes a manual, parallel or alternative process used to manage a workflow, checking or escalation problem. | Formal standard operating procedures functioning as intended. | Manual workaround; parallel process; alternative checking process; informal adaptation. |
| Amendment authority | Practical ability to alter, correct, reject or delay an output, document or decision. | A contributor can edit generated text, stop discharge, reject a summary or require reconsideration. | Merely noticing an error without power to change the outcome. | Can edit; refuse; reject; hold discharge; change order; send back. |
| Restricted authority | Recognition of a problem without sufficient power to change the relevant output, decision or organisational condition. | Frontline staff identify risk but describe being overruled, unable to stop a process or lacking decision rights. | Ordinary disagreement where escalation and reconsideration remain effective. | Overruled; cannot stop it; management decides; no authority; decision made elsewhere. |
| Escalation | Movement of a concern to a person or structure with greater authority or capacity to act. | Contributors contact senior clinicians, managers, social work, governance, IT, risk, or other decision-makers. | Routine referral not prompted by a concern or need for higher authority. | Escalate; call attending; report; raise with manager; incident process. |
| Professional scope | Boundaries of what a role is authorised, competent or expected to do. | Discussion concerns role limits, supervision, delegation, prescribing, signing, or scope-related accountability. | General hierarchy with no reference to role boundaries or permitted practice. | Within scope; outside scope; requires attending; nurse vs physician role. |
| System-level control | Authority over recurrent defects, workflow rules, staffing assumptions, system operation or continued deployment. | Discussion shifts from correcting an individual output to changing recurring organisational or system-level conditions. | Output-level editing or correction by an individual clinician only. | Recurrent defects; workflow rules; staffing assumptions; continued deployment; system-level intervention. |
| Cases of adequate capacity | Counter-cases where assigned responsibility is matched by sufficient information, time, expertise and/or authority. | Contributors describe workable review, accessible sources, manageable checking, effective escalation or clear role structures. | Absence of a problem without evidence that capacity was actually adequate. | Enough time; clear escalation; source available; can correct; roles work as intended. |

## Table S5b. Interactional coding layer

| Code | Operational definition | Include when | Exclude / distinguish from | Analytic indicators |
| --- | --- | --- | --- | --- |
| Agreement / reinforcement | A reply supports, confirms or strengthens a prior claim. | Explicit endorsement, corroborating example or extension of the same position. | Neutral continuation without evaluative support. | Yes; exactly; same here; confirming example. |
| Challenge / rebuttal | A reply disputes or rejects a prior claim. | Direct disagreement, correction or counter-argument. | Simple clarification or qualification without rejection. | No; that is not how it works; correction; opposing account. |
| Qualification | A reply narrows, conditions or adds limits to a prior claim. | The responder agrees partly but specifies exceptions, conditions or context. | Full agreement or full rejection. | Depends; in my setting; only if; except when. |
| Responsibility redirection | A reply shifts responsibility from one actor or level to another. | A commenter says another profession, team, organisation or family should own the task. | Shared responsibility where no shift occurs. | That belongs to X; management owns this; family responsibility. |
| Knowledge challenge | A reply questions the adequacy, accuracy or legitimacy of another participant's knowledge. | The responder disputes expertise, factual basis, interpretation or access to relevant information. | Disagreement about values without a knowledge claim. | You do not have the full chart; inaccurate; not clinically informed. |
| Authority claim | A reply invokes role, policy, hierarchy or decision rights to justify who may act. | Reference to attending authority, policy, scope, managerial power, legal authority or formal responsibility. | Expertise claim without decision rights. | Per policy; attending decides; manager can override; within my scope. |
| Boundary defence | A reply protects a professional, ethical or role boundary. | A contributor states what they will not do, cannot be expected to do, or should remain outside their role. | General refusal not tied to role/boundary logic. | Not within scope; I will not sign; this crosses a boundary. |
| Elaboration | A reply adds detail, explanation or an example without materially changing the prior position. | Additional context, workflow detail, narrative evidence or clarification. | Simple repetition with no added content. | For example; in our unit; here is what happens next. |
| Workaround proposal | A reply suggests an informal practical solution to a workflow or oversight problem. | Manual checks, alternate documentation, custom prompts, parallel processes, informal escalation. | Formal policy response already embedded in routine process. | Keep your own note; manually verify; use a separate list. |
| Conversational settlement | A sequence reaches an apparent practical resolution or shared understanding. | Participants converge on an explanation, action or boundary. | Thread simply ends without resolution. | Consensus emerges; agreed action; accepted clarification. |
| Continuing disagreement | Competing interpretations remain unresolved across replies. | Participants continue to contest facts, responsibility, authority or meaning. | Single disagreement later resolved. | Back-and-forth dispute; no consensus. |
| Counterexample | A reply supplies a case that contradicts or limits a developing generalisation. | Example shows adequate capacity, effective authority, benefit, or a different mechanism. | Unrelated anecdote that does not bear on the claim. | Works well here; source access makes checking easy; escalation solved it. |

## Negative-case and counter-case rule

An AI error, workload complaint, difficult discharge or requirement for checking was not sufficient on its own to constitute oversight asymmetry. Interpretation required an identifiable responsibility to be constructed as disproportionate to the information, time or authority available to discharge it. Where an actor lacked final decision rights but described an effective escalation route, the arrangement was read as distributed oversight. Accounts showing adequate source access, manageable verification, sufficient time or effective authority were retained as counterexamples and used to refine theme boundaries.

# S6. Relationship to the authors’ previous Reddit studies

| Characteristic | Reference 40 | Reference 41 | Present study |
| --- | --- | --- | --- |
| Period covered | 1 January 2023 to 31 December 2025 | October 2020 to February 2026 | Threads posted 2021 to 25 April 2026, collected 3 March to 16 May 2026 |
| Communities | r/medicine, r/Residency, r/FamilyMedicine and r/emergencymedicine | Eight clinician-oriented communities | 16 predefined communities, five contributing retained threads |
| Retrieval | Separate retrieval strategy | Separate retrieval strategy | Separate 90-query strategy (S1, S2) |
| Corpus | 162 threads, 952 eligible documents, 2,267 incident segments | 120 threads, 484 relevant comments | 64 retained threads, 49 used in the final themes |
| Unit of analysis | Incident segment | Relevant comment within thread | Retrieved thread |
| Analytic focus | Socio-technical mechanisms behind AI scribe documentation failures | Patient-facing AI-generated documentation, accountability, privacy and record integrity | Mismatch between assigned verification responsibility and reviewer capacity, using neonatal discharge as a test context |

All material retained in the present study was screened and coded afresh against the present research question. No quotation, prepared paraphrase, published thematic label or previous coding assignment from references 40 or 41 was carried forward as evidence. Because those publications do not include complete inventories of Reddit submission identifiers, exact identifier-level overlap cannot be reconstructed from the publications alone. The screening workbook for the present study, which contains the Reddit submission identifier of every candidate thread, is available to the editor and reviewers on request, so that any overlap can be checked directly.

# S7. SRQR compliance map (updated)

| Item | SRQR topic | Location | How addressed |
| --- | --- | --- | --- |
| 1 | Title | Main manuscript, title and first page | The title identifies oversight asymmetry, AI-assisted clinical documentation, the comparative-abductive design and neonatal discharge as a test context. |
| 2 | Abstract | Main manuscript, Abstract | The abstract reports the governance problem, design, 64 retained threads with comment trees retrieved under prespecified parameters, 2,848 comments from 1,723 pseudonymised accounts, the 49 contributory threads, principal findings stated as patterns and propositions, and prospective implications. |
| 3 | Problem formulation | Main manuscript, Introduction | Introduction establishes the governance problem, verification demand and the gap concerning responsibility-capacity mismatch. |
| 4 | Purpose or research question | Main manuscript, Abstract and Introduction | The study aim is stated in the abstract and the research question is stated in the Introduction. |
| 5 | Qualitative approach and research paradigm | Main manuscript, Materials and methods, Research design and analytical orientation | Comparative-abductive, netnographically informed qualitative design, reflexive thematic analysis, contextualist and interpretivist orientation are specified. |
| 6 | Researcher characteristics and reflexivity | Main manuscript, Materials and methods, Reflexivity and analytic quality; Supplementary S5 | The Reflexivity and analytic quality section reports circularity risk, researcher roles, a researcher positionality statement covering clinical, informatics and prior research backgrounds, and responses to disagreement. |
| 7 | Context | Main manuscript, Introduction and Materials and methods | Neonatal discharge context, Reddit communities, period represented and the rationale for the online discussion setting are described. |
| 8 | Sampling strategy | Main manuscript, Materials and methods; Supplementary S1 to S4 | Purposive community selection, the 90-query set, bounded retrieval, the revision of the research question before full coding, screening and the post-screening audit are reported, with a study-flow diagram (Figure S1). |
| 9 | Ethical issues | Main manuscript, Materials and methods, Ethical handling of public online material; Ethics statement | The Ethics section reports the University of Staffordshire Level 1 Ethics Disclaimer (DEF-A041261N-001), pseudonymisation, paraphrase-only reporting with trace-back checking and data-protection handling. |
| 10 | Data collection methods | Main manuscript, Materials and methods; Supplementary S1 to S4 | Reddit Data API retrieval through PRAW, query combinations, extraction limits, deduplication, dates of the retained material and screening are described (S1, S2). |
| 11 | Data collection instruments and technologies | Main manuscript, Materials and methods; Supplementary S1 | The query-generation script and retrieval configuration are reported in S1. |
| 12 | Units of study | Main manuscript, Materials and methods | The retrieved Reddit thread is the unit of analysis, with comments and replies as embedded meaning units. |
| 13 | Data processing | Main manuscript, Materials and methods; Supplementary S1 to S4 | Deduplication, hierarchy reconstruction, handling of deleted accounts, pseudonymisation and stream allocation are described. |
| 14 | Data analysis | Main manuscript, Materials and methods; Supplementary S5 | Open first-cycle coding, interactional coding, recursive theme development and subsequent abductive interrogation through five sensitising dimensions are detailed. |
| 15 | Techniques to support trustworthiness | Main manuscript, Materials and methods, Reflexivity and analytic quality; Supplementary S5 | Negative and boundary cases, worked contrasts, an audit trail, contributor-concentration review and reflexive memoing are reported. |
| 16 | Synthesis and interpretation | Main manuscript, Results and Discussion | Four themes are synthesised, followed by the oversight-asymmetry interpretation and governance propositions. |
| 17 | Links to empirical data | Main manuscript, Results | Findings are reported as paraphrase with internal thread identifiers, evidence stream and community, linked to the inventory in S4. |
| 18 | Integration with prior work and implications | Main manuscript, Discussion | The Discussion relates findings to meaningful human control, responsibility gaps, verification complexity and AI-assisted documentation, while Figure 1 synthesises the prospective governance mechanism and intervention points for responsible digital paediatrics. |
| 19 | Limitations | Main manuscript, Discussion, Limitations; Table 7 | The Limitations subsection and Table 7 address platform, sampling, jurisdictional, temporal, evidence-stream and inferential limitations. |
| 20 | Conflicts of interest | Main manuscript, Conflict of interest | The Conflict of interest statement reports that the authors declare no commercial or financial relationships that could be construed as a potential conflict of interest. |
| 21 | Funding | Main manuscript, Funding | The Funding statement reports financial support from the Local Maternity and Neonatal System (LMNS) for Leicester, Leicestershire and Rutland, United Kingdom. |
